# Endoplasmic reticulum stress at the forefront of fatty liver diseases and cancer

**DOI:** 10.1016/j.pharmr.2025.100096

**Published:** 2025-10-14

**Authors:** Michael Karin, Ju Youn Kim

**Affiliations:** 1 Center for Metabolic and Liver Diseases, Sanford Burnham Prebys Medical Discovery Institute, La Jolla, California; 2 Department of Molecular and Life Science, College of Advanced Technology and Convergence, Hanyang University ERICA, Ansan, Republic of Korea

## Abstract

The endoplasmic reticulum (ER) is a dynamic membranous organelle that accounts for nearly half of the total membrane content in hepatocytes and serves as a central hub for protein folding and lipid biosynthesis. Given the liver’s essential functions in protein production and secretion, lipid handling, and xenobiotic metabolism, hepatocyte ER homeostasis is essential for systemic metabolic control and health. Metabolic dysfunction-associated steatotic liver disease, which affects nearly 30% of the global population, is strongly linked to hepatic ER stress. Accumulating evidence highlights the unfolded protein response (UPR) as a key mechanistic regulator that integrates proteostasis and metabolic stress, thereby influencing disease progression from simple steatosis to inflammation-driven metabolic dysfunction-associated steatohepatitis (MASH). More recently, ER stress has also been implicated as a driver of MASH-related hepatocellular carcinoma, the most common primary liver cancer. In this review, we provide a comprehensive overview of the dynamic roles of the UPR and ER stress in hepatocytes, with particular emphasis on mechanistic insights derived from murine models of MASH-related hepatocellular carcinoma. We also summarize the current animal models of MASH that depend on hepatic ER stress. Finally, we discuss therapeutic candidates for MASH treatment, whose mechanisms of action involve ER stress and the UPR.

## 1. Introduction

The endoplasmic reticulum (ER) is a dynamic membranous organelle divided into 2 domains: rough ER (RER) and smooth ER (SER). The RER, defined by ribosome-studded membranes, directs the synthesis of secretory proteins, integral membrane proteins, and luminal resident organellar proteins.^1^ In contrast, the SER lacks ribosomes but is enriched in enzymes required for lipid biosynthesis and xenobiotic detoxification.^1^ Through these specialized subdomains, the ER acts as a signaling hub that senses disrupted proteostasis, lipid imbalance, or impaired detoxification and transmits related stress signals to gene regulatory pathways via ER membrane-bound stress sensors.^2^ Therefore, ER plays a pivotal role in alleviating cellular stress, maintaining homeostasis, and restoring normal liver function.

Protein maturation in the ER is monitored by a stringent quality-control system. Misfolded polypeptides are recognized by ER-resident chaperones and folding enzymes, which promote refolding and stabilization.^3^ When the demand for protein folding exceeds the ER capacity, the unfolded protein response (UPR) is activated. This evolutionarily conserved signaling cascade is initiated by 3 sensors, inositol-requiring enzyme 1 (IRE1), protein kinase RNA-like ER kinase (PERK), and activating transcription factor 6 (ATF6).^4^ UPR activation initiates transcriptional and translational programs that suppress global protein synthesis, expand ER folding capacity, and increase ER volume. Terminally misfolded proteins are eliminated through ER-associated degradation (ERAD), which delivers misfolded and aggregated substrates to the proteasome, thereby recycling amino acids.^5^ Although the primary function of the UPR is to restore ER proteostasis and maintain cellular viability, chronic or unresolved ER stress shifts the response toward apoptotic cell death.^6^

Typical ER stress inducers are multiple metabolic stressors, nutrient excess, lipotoxicity, and oxidative stress, all of which are commonly observed in metabolic dysfunction-associated steatotic liver disease (MASLD), an obesity-driven global liver disorder. Accumulating evidence identifies ER stress as a central driver of MASLD progression and a critical contributor to the pathogenesis of MASH and MASH-related hepatocellular carcinoma (HCC).

In this review, we delineate the molecular mechanisms through which ER stress drives MASLD pathogenesis, with particular emphasis on the distinct and overlapping roles of the 3 UPR sensors across disease stages. We also provide a systematic evaluation of animal models, with specific attention to their relevance to human pathogenic progression in MASH, and critically examine emerging therapeutic strategies that target metabolic stress, highlighting their potential as preventive interventions against MASH-related HCC.

## II. Metabolic dysfunction-associated steatotic liver disease and metabolic dysfunction-associated steatohepatitis

The previously used term of nonalcoholic fatty liver disease (NAFLD) was coined in 1980 to classify patients in whom liver fat accumulation (hepatosteatosis) is driven by obesity and excessive intake of energy-dense diets in the absence of significant alcohol consumption. By definition, NAFLD diagnosis requires the exclusion of secondary causes of steatosis, such as alcohol abuse or viral hepatitis.^7^ However, more than 3 decades of clinical experience have revealed significant diagnostic limitations. Patients with NAFLD frequently present overlapping features with NAFLD-associated comorbidities, including hepatosteatosis and portal inflammation, which complicates both accurate diagnosis and effective management. These challenges have prompted the redefinition of NAFLD in a manner that better reflects clinical reality. Moreover, the terminology “nonalcoholic” and “fatty” has been criticized as stigmatizing. To address these concerns, an international panel of hepatologists, gastroenterologists, endocrinologists, healthcare providers, and patient advocates convened to re-evaluate the nomenclature, resulting in the new term MASLD.^8^ The diagnostic criteria for MASLD require the presence of hepatic steatosis in combination with at least 1 cardiometabolic risk factor, thereby shifting the framework toward pathophysiological mechanisms rather than exclusion-based definitions.

The clinical spectrum of MASLD mirrors that of its predecessor, NAFLD, encompassing a continuum of pathological conditions with varying severity. This includes metabolic dysfunction-associated steatotic liver (MASL) and metabolic dysfunction-associated steatohepatitis (MASH).^7^ MASL refers to individuals with hepatic lipid accumulation in more than 5% of hepatocytes, without significant hepatocellular injury or inflammation. By contrast, MASH is characterized by hepatosteatosis accompanied by active necroinflammation, including hepatocellular ballooning, lobular inflammation, and variable degrees of fibrosis.^7^ MASH represents a silent but progressive disorder that can advance to cirrhosis, a life-threatening stage that often necessitates disease-modifying interventions, including liver transplantation.^9^ Reflecting the increasing prevalence of alcohol consumption as a common social behavior, recent updates to the MASLD definition now include individuals with moderate alcohol intake, defined as <20 g/day for women and <30 g/day for men. Furthermore, a new subcategory, metabolic dysfunction-associated alcohol-related liver disease, has been proposed to identify individuals whose alcohol consumption (20—50 g/day for women; 30—60 g/day for men) significantly contributes to disease progression.^8^ As MASLD progresses to more advanced stages, patients encounter an increased risk of cirrhosis and HCC, referred to as MASH-related HCC.^10^

### A. Metabolic dysfunction-associated steatohepatitis-related hepatocellular carcinoma

HCC is the most common form of primary liver cancer and ranks as the fourth leading cause of cancer-related death worldwide.^11^ HCC pathogenesis is driven by chronic liver injury and inflammation, which promote sustained necroinflammatory signaling and compensatory hepatocyte regeneration.^12^ Historically, environmental and lifestyle-related exposures such as excessive alcohol intake, toxicant exposure, and viral hepatitis were the dominant risk factors, with marked geographical variation in incidence.^13^ In recent years, however, the increased consumption of energy-dense obesogenic diets has contributed to a sharp global increase in HCC incidence, independent of regional differences.^14^ As a consequence, HCC-related mortality increased by 43% in the United States between 2000 and 2006, and the World Health Organization projects that by 2030, over 1 million individuals will die of HCC, most of them MASH-related.^11^

Current epidemiological studies estimate that MASH accounts for approximately 10% of global HCC cases,^15,16^ but as mentioned above, this is certain to increase as there is a delay between the onset of MASH and its progression to HCC. Among the diverse clinical features of MASH, hepatic fibrosis appears to be the most critical determinant of cancer risk, with fibrosis severity strongly correlating with increased HCC incidence.^17^ Indeed, fibrosis is considered the strongest predictor of liver-related morbidity and mortality,^18^ but this could be due to a strong link between fibrosis and inflammation, a well recognized promoter of HCC.^12^ Individuals with advanced fibrosis (stage ≥3, F3) and a MASLD activity score (MAS) of 5—8 face a 15-fold higher risk of HCC-related mortality.^19^ Nevertheless, a subset of MASH patients develops HCC in the absence of advanced fibrosis or cirrhosis,^20^ underscoring the heterogeneous and multifactorial nature of MASLD-related hepatocarcinogenesis in this population and its dependence on steatohepatitis rather than fibrosis.

### B. Obesity as a trigger of metabolic dysfunction-associated steatohepatitis-associated hepatocellular carcinoma

The continued rise in global obesity rates is projected to proportionally increase the incidence of MASH and MASH-related HCC, alongside other obesity-associated comorbidities.^21^ More concerning than the obesity epidemic itself is the recognition that obesity functions as an independent risk factor for MASH-related HCC. Clinical evidence shows that obesity accelerates MASH progression, increases the likelihood of cirrhosis, and markedly raises the risk of MASH-associated HCC.^22^ Epidemiological studies further demonstrate that morbid obesity (body mass index [BMI] >40) significantly elevates the risk of HCC-related death in both men and women.^23^ Although the magnitude varies, childhood and adolescent obesity are also positively correlated with future cancer morbidity.^24^ In particular, overweight status itself predisposes patients with liver dysfunction to septal fibrosis, thereby elevating the risk of MASLD-related liver transplantation and HCC.^25,26^ Mouse studies also have shown that diet-induced obesity greatly enhanced HCC development in the presence of hepatic ER stress with the underlying mechanism being increased production of the proinflammatory cytokines, tumor necrosis factor (TNF), and interleukin-6 (IL-6).^27,28^

Notably, however, lean patients, defined as those with a BMI <25 in Western populations or a BMI <23 in Asian populations, can also develop MASLD. This phenotype, termed “MASLD in lean individuals,” is frequently associated with significant metabolic abnormalities, including hyperglycemia, hypertension, and hyperinsulinemia.^29^ These seemingly contradictory clinical findings strongly suggest that excessive fat mass is not the principal causal factor in MASLD pathogenesis. Thus, a more comprehensive understanding will require integrated analyses that combine metabolic parameters, genetic risk factors, and epidemiological data, potentially aided by machine learning approaches, to elucidate the molecular mechanisms driving MASH progression to HCC.

## III. Endoplasmic reticulum stress and metabolic dysfunction-associated steatotic liver disease

**Fig. 1.**
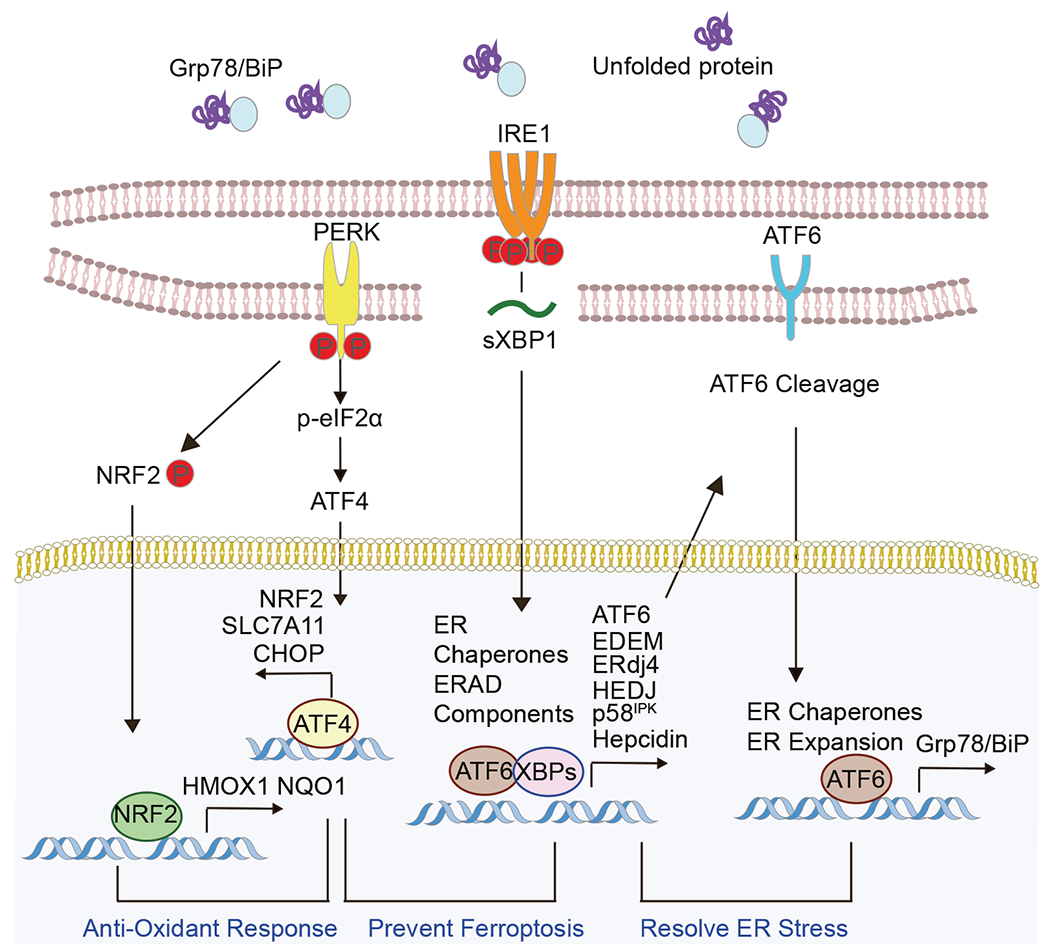
UPR actions under normal conditions or in MASL hepatocytes. The UPR acts cooperatively to restore ER homeostasis in hepatocytes. P, phosphorylation.

ER stress is triggered by alterations in redox balance and in response to cellular metabolic stress, including increased amounts of membrane-perturbing nonesterified saturated fatty acids (NESFA) and the accumulation of misfolded proteins.^30^ These perturbations disrupt ER homeostasis and activate the UPR.^30,31^ The 3 principal UPR sensors, IRE1, ATF6, and PERK, exert cooperative yet distinct functions that collectively restore proteostasis and resolve ER stress (Fig. 1).

### A. IRE1

IRE1 is the most evolutionarily conserved UPR sensor. Unlike yeast, which harbors a single isoform (Ire1p), mammals express 2 paralogs, IRE1*α* and IRE1*β*.^32,33^ Given that IRE1*α* is the predominant isoform in the liver, this review will primarily focus on its role in MASH unless otherwise noted.

Structurally, IRE1 isoforms are type I transmembrane proteins composed of an N-terminal luminal domain, a transmembrane region, and a ~130 amino acid—long cytosolic tail.^34^ The luminal domain contains a hydrophilic stretch with 4 cysteine residues and 4 N-glycosylation sites.^34^ These elements enable IRE1 to act as a sensitive monitor of misfolded protein load within the ER, transducing this stress signal to the nucleus and leading to the transcriptional activation of ER-related genes.^32^ Signal transduction requires IRE1 oligomerization, which juxtaposes its cytosolic Ser/Thr kinase and endoribonuclease (RNase) domains, a process regulated by the ER chaperone 78 kDa glucose-regulated protein (Grp78/BiP). Under homeostatic conditions, Grp78/BiP maintains it in an inactive state by preventing oligomerization.^35^ During ER stress, Grp78/BiP dissociates from IRE1 to preferentially bind unfolded or misfolded proteins, thereby allowing IRE1 to oligomerize^36^ and undergo transautophosphorylation at Ser724, which is essential for activating IRE1’s RNase activity.^37^

Although IRE1 oligomerization is considered essential for activating its RNase function, the precise molecular events linking oligomerization to RNase activation remain incompletely understood. Upon activation, IRE1 assembles into discrete foci along the ER membrane.^37^ Interestingly, mutations that disrupt the luminal Grp78/BiP-binding domain abolish RNase activity despite the fact that mutant IRE1 still forms foci, suggesting that oligomerization alone is insufficient.^37^ Structural analyses further revealed that the kinase domain in the IRE1 cytosolic tail is intimately aligned with the RNase domain,^38^ and mutations that eliminate kinase activity concurrently impair RNase function.^39^ Collectively, these findings underscore the importance of both structural integrity and kinase activity in mediating IRE1’s RNase function.^38–40^ Notably, IRE1 exists in both dimeric and oligomeric conformations, and these structural states appear to dictate substrate specificity.^41^ Oligomeric IRE1 preferentially promotes noncanonical mRNA splicing, whereas dimeric IRE1 favors regulated IRE1-dependent mRNA decay (RIDD).^41^ Thus, IRE1 activation is likely determined by a combination of upstream ER stress signals and the architectural context of IRE1 assembly.

IRE1’s endoribonuclease domain targets 2 classes of RNA substrates: it catalyzes the unconventional splicing of specific mRNAs and cleaves the selected microRNAs.^42,43^ The most well characterized mRNA substrate codes for the X-box binding protein 1 (XBP1), a basic leucine zipper (bZIP) transcription factor. Upon activation, IRE1 excises a 26-nucleotide intron from unspliced XBP1 mRNA by recognizing 2 conserved sites, each containing 1 cytosine and 2 guanine residues. These cleavage events generate 2 RNA fragments that are subsequently ligated to produce spliced XBP1 mRNA. These splicing and religation processes introduce a translational frameshift, resulting in a protein with an additional 104 amino acids. Importantly, only spliced XBP1 functions as an active transcription factor, whereas unspliced XBP1 lacks transcriptional stimulatory activity.^42^

Given that XBP1 is essential for the differentiation of plasma cells, where ER-Golgi—mediated secretion is essential for antibody production,^44^ hepatic XBP1 has been suggested to regulate ER homeostasis. Consistently, microarray analysis of XBP1-deficient cells revealed that expression of several genes coding for ER-resident proteins, including *ERdj4*, *p58^IPK^*, *EDEM1*, and *HEDJ*, is critically dependent on XBP1.^45,46^ In addition, XBP1 regulates genes involved in ERAD,^45,46^ ER chaperones,^47^ and ER biogenesis,^48^ indicating that IRE1-induced XBP1 splicing plays a central role in maintaining hepatic proteostasis.

RIDD represents another critical facet of IRE1 function, essential for resolving ER stress. A remarkable feature of IRE1 is its ability to selectively recognize RIDD substrates in response to specific upstream stress signals.^49^ Tam et al^41^ demonstrated that although higher-order IRE1 oligomerization is necessary for mRNA splicing, RIDD activity is mediated by a distinct domain within the IRE1 dimer and does not require higher-order assembly. Notably, 1 class of RIDD substrates includes microRNAs, which are implicated in miRNA-regulated apoptosis.^41,49^ In mouse embryonic fibroblasts subjected to ER stress, IRE1 was found to cleave miR-17, miR-34, and miR-125 using a lysine residue located at position 906. These miRNAs typically bind to the 3′ untranslated region of caspase-2 (Casp2) mRNA, and their cleavage leads to the derepression and subsequent upregulation of Casp2 translation.^43^

### B. ATF6

By screening putative binding partners of the ER stress-response element (ERSE), the human transcription factor ATF6 was identified as another critical mediator of the UPR/ER stress response.^50,51^ ATF6 is also a bZIP transcription factor and a type II transmembrane glycoprotein, with its ER anchoring transmembrane domain located near the center.^51^ Similar to IRE1 activation, the luminal domain of ATF6 binds to Grp78/BiP, which masks its C-terminal translocation signal required for Golgi translocation and subsequent site-1 protease (S1P) and site-2 protease—mediated proteolytic cleavage, called regulated intramembrane proteolysis (RIP). Upon ER stress, Grp78/BiP dissociates from ATF6, exposing the Golgi translocation signal.^52^ Once ATF6 moves to the Golgi apparatus, S1P initiates its proteolytic activation,^52,53^ resulting in the formation of either homodimers or heterodimers, which coordinate the adaptive responses to ER stress.^47^ Following activation, the processed form of ATF6 exhibits high-affinity for ERSE, particularly as a heterodimer with nuclear factor Y (NF-Y), which drives strong upregulation of major ER chaperones such as Grp78/BiP, Grp94, and calreticulin.^50^ Consistently, ATF6-deficient cells fail to induce ER chaperones and become susceptible to ER stress-induced apoptosis.^50,54^ Moreover, ATF6 regulates a broad set of ERAD components, including *Edem1*, *Hrd1*, and *Herpud1*,^47,50^ establishing ATF6 as a major transcriptional activator of the ER protein-folding machinery.

Sequence analysis of the *XBP1* promoter revealed the presence of a UPR element cis-acting motif to which ATF6 binds.^47^ Consistently, ATF6 promotes transcription of *XBP1* mRNA,^55^ which then undergoes IRE1-mediated splicing. Remarkably, IRE1 alone is insufficient to efficiently cleave XBP1. Rather, ATF6-induced transcription of XBP1 is required to ensure adequate substrate availability for IRE1-dependent splicing and functional activation.^55^ This highlights that ATF6 not only functions as a transcription factor but also plays an indispensable role in facilitating IRE1-dependent XBP1 activation. Supporting this, both ATF6 and XBP1, as members of the bZIP transcription factor family, can form heterodimers that cooperatively enhance XBP1 expression and activity.^47^

ER membrane expansion is a critical adaptive response that alleviates ER stress by expanding the capacity to hold ER chaperones. In NIH-3T3 cells, transfection with an ATF6-activating mutant [ATF6(1—373)] followed by electron microscopy revealed pronounced RER enlargement.^48^ Notably, this effect was specific to ATF6 activation and was not observed with activation of other UPR sensors, indicating that ATF6 directly drives ER membrane expansion.^48^ Mechanistically, ER membrane biogenesis requires the phospholipid phosphatidylcholine, synthesized via the Kennedy pathway, which involves enzymes such as choline kinase and choline phosphotransferase.^56^ Cells expressing ATF6 (1—373) exhibit robust upregulation of choline kinase and choline phosphotransferase, leading to enhanced de novo synthesis of phospholipid phosphatidylcholine.^48,57^ Together, these findings reinforce the notion that ATF6 promotes ER biogenesis and remodeling by regulating lipid biosynthesis pathways.

### C. PERK

ER protein folding is tightly coupled to the translational output of ribosomes. Consequently, attenuation of protein synthesis under ER stress provides a key adaptive strategy that mitigates misfolding burden. Among the UPR sensors, PERK was identified as the pivotal component of the translational regulator.^58^ Interestingly, the N-terminal luminal domain of PERK shares approximately 20% sequence homology with IRE1, suggesting a capacity to sense misfolded proteins within the ER lumen. The C-terminal cytosolic region of PERK contains a Ser/Thr kinase domain that shares ~40% sequence identity with that of dsRNA-induced protein kinase.^58^ Upon sensing ER stress caused by protein misfolding, PERK transduces this signal through its cytosolic kinase domain, initiating phosphorylation of downstream effectors critical for translational control, including the *α*-subunit of eukaryotic translation initiation factor 2 (eIF2*α*), which regulates protein synthesis under metabolic stress.^59^ During translation initiation, eIF2*α* binds guanosine 5′-triphosphate (GTP) and initiator methionine transfer RNA to form the 43S preinitiation complex with the 40S ribosome subunit.^59^ Upon recruitment of the 60S subunit and mRNA, GTP bound to eIF2*α* is hydrolyzed and exchanged for guanosine diphosphate (GDP) by the guanine nucleotide exchange factor eIF2B, enabling recycling of eIF2*α* for subsequent rounds of translational initiation.^59^ This cycle is tightly regulated by post-translational modifications, including phosphorylation of eIF2*α* at Ser51, which markedly increases its affinity for eIF2B, sequestering the exchange factor and preventing GDP-GTP replacement. When the pool of phosphorylated eIF2*α* exceeds that of available eIF2B, the intracellular pool of GDP-eIF2*α* is reduced, and ribosomal protein translation is eventually attenuated.^60^ Of note, PERK directly phosphorylates eIF2*α*, thereby inhibiting protein synthesis.^61^ Mutation of a critical lysine residue (Lys618) within the catalytic domain abolishes PERK’s kinase activity and fails to inhibit global protein translation, highlighting the role of PERK in translational regulation under ER stress.^58^ PERK is abundantly expressed in secretory organs, including the pancreas, liver, spleen, and thymus. Although PERK-deficient mice appear phenotypically normal at birth, they develop spontaneous diabetes with age, attributed to defective insulin secretion and unresolved *β*-cell ER stress.^62^

Although global suppression of protein synthesis is essential to alleviate the ER protein misfolding burden, expression of specific ER proteins remains indispensable for the adaptive responses.^60^ This paradox was resolved by the discovery of eIF2*α* as a central mediator that balances these opposing demands. PERK-mediated phosphorylation of eIF2*α* suppresses overall translation but simultaneously permits selective translation of the bZIP activating transcription factor 4 (ATF4), a master regulator of the integrated stress response.^63,64^ Another ER stress-relevant bZIP transcription factor is CHOP, whose induction drives the expression of GADD34, a regulatory subunit of protein phosphatase 1 that mediates dephosphorylation of eIF2*α*.^65^ Once ATF4 is activated, it upregulates CHOP to induce GADD34 expression. GADD34-mediated dephosphorylation of eIF2*α* relieves cells from translational suppression, thereby restoring global protein synthesis.^66^ However, under conditions of persistent ER stress, resumption of translation reintroduces nascent peptides into an already burdened ER, further aggravating proteotoxic stress and ultimately triggering ER stress-induced apoptosis. This feedback mechanism exemplifies how ER stress employs its own negative regulatory loop to determine cell fate, with the PERK-ATF4-CHOP axis serving as a switch between adaptive recovery and apoptotic commitment.^64,66^

## IV. Role of UPR in metabolic dysfunction-associated steatohepatitis progression

MASH is a complex and heterogeneous metabolic disorder, the progression of which is influenced by various etiologies.^67^ However, not much is known about the MASH progression to HCC. The deposition of harmful lipid species into cellular organelles other than lipid droplets, such as the ER and mitochondria, induces stress responses that lead to hepatocyte death and result in the activation of liver-resident and circulating immune cells.^68–70^ On the other hand, hepatic cholesterol derivatives, such as bile acids (BA), play a key role in regulating intestinal lipid metabolism and absorption, and their impaired regulation has been frequently observed in MASH-afflicted livers.^71,72^ Genome-wide association studies using biopsies from patients with either early MASLD or advanced MASH patients had revealed several mutated or polymorphic genes in patients with MASH, including *PNPLA3*, *TM6SF2*, *MBOAT7*, and *HSD17B13*. Among these genes, the *PNPLA3* variant, *PNPLA3 (I148M)*, is strongly associated with the severity of liver damage and MASH progression.^73,74^ Mutational profiling of MASH-related HCC has further revealed that *TERT*, *CTNNB1* , *TP53*, and *ACVR2A* are among the most frequently altered genes. Notably, *ACVR2A* represents a unique mutational signature exclusive to MASH-related HCC, distinguishing it from HCC arising from other etiologies.^20^ Additionally, dietary fructose, a highly lipogenic carbohydrate, was found to increase intestinal permeability^75^ and cause low-grade endotoxemia that contributes to MASH development.^76^ In addition to endotoxin, several other metabolites produced by intestinal microbes were suggested as critical contributors to MASH progression.^76–78^

**Fig. 2.**
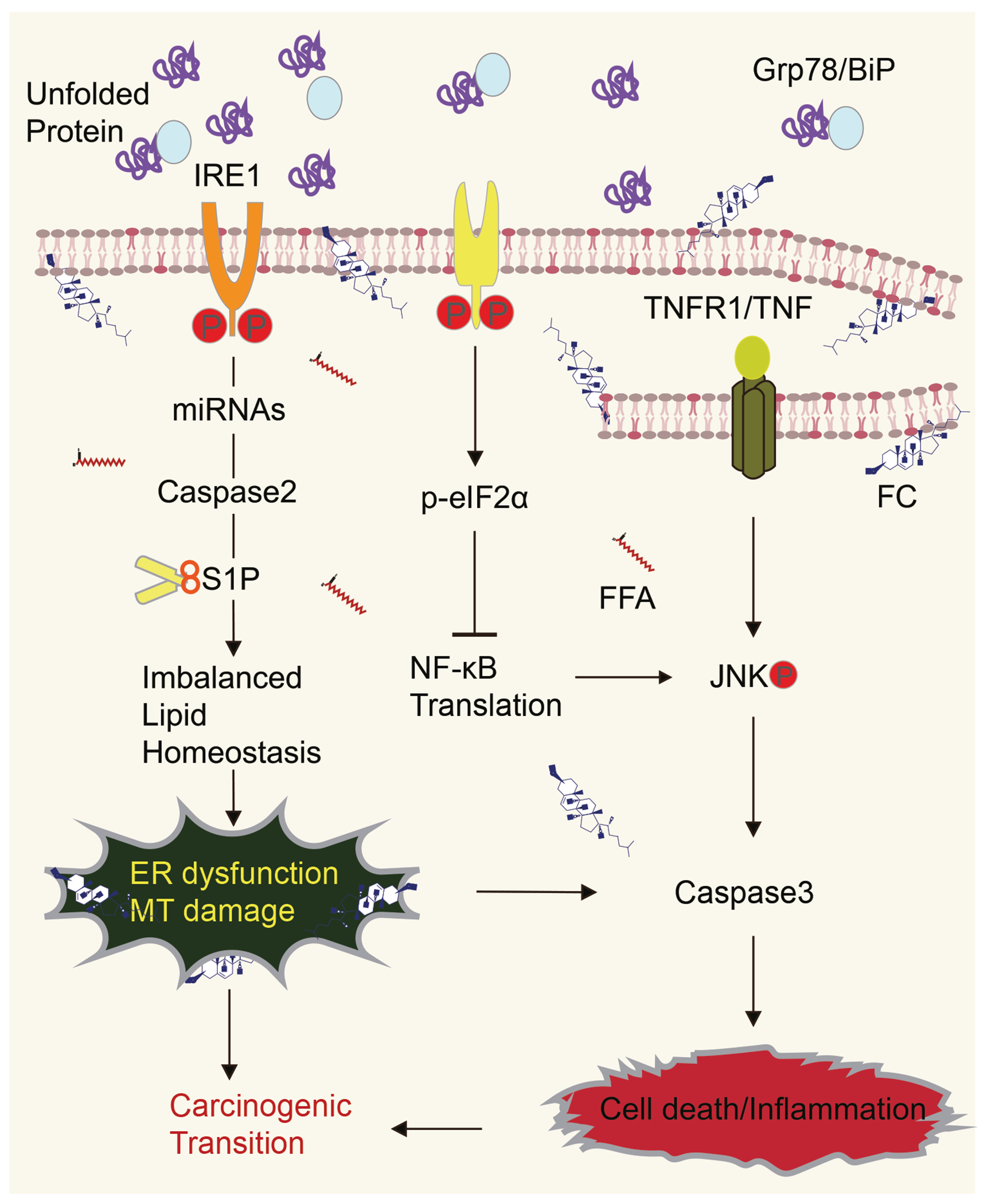
UPR and TNFR1 signaling synergistically trigger cell death under lipotoxic stress by engaging apoptotic pathway and organellar dysfunctions. ER, endoplasmic reticulum; FC, free cholesterol; FFA, free fatty acids; MT, mitochondria; P, phosphorylation.

Taken together, these studies suggest that multiple molecular pathways are involved in MASH pathogenesis and its progression to HCC,^67^ including ER stress^79^ on which this review is focused (Fig. 2).

### A. Lipotoxicity is central to metabolic dysfunction-associated steatohepatitis-related hepatocellular carcinoma progression

Liver steatosis can arise from 3 sources: free fatty acids (FFA) and NESFA that spill over from adipose fat depots or are generated by hepatic de novo lipogenesis (DNL) and dietary lipids. Donnelly et al^80^ estimated the contribution of each source in patients with MASLD and found that dietary lipids and adipose tissue spillover account for 16% and 60%, respectively, whereas hepatic DNL accounts for 24% of the lipids accumulated in the liver. However, this assertion is not fully supported by experimental data. For instance, overexpression of diacylglycerol acyltransferase 2 (DGAT2), which catalyzes the formation of an ester linkage between fatty acyl-CoA and diacylglycerol (DAG),^81^ in mouse hepatocytes resulted in massive liver steatosis.^82^ Nonetheless, the steatotic liver remained insulin-sensitive and glucose-tolerant, suggesting that hepatic triglyceride (TG) deposition in lipid droplets protects the liver from insulin resistance and metabolic complications, as opposed to membrane incorporation of NESFAs.^82^ Moreover, inhibition of DGAT2 expression with antisense oligonucleotides in methionine-choline-deficient diet (MCD) fed mice resulted in a significant reduction in steatosis but also led to markedly increased FFA and oxidative stress, which in turn aggravated liver ER stress and injury.^83^ Overexpression of DGAT1 in adipocytes prevented FFA-induced ER stress and cell death, protecting the mice from liver steatosis and the metabolic syndrome.^84,85^ Considering these findings, it was suggested that liver steatosis alone cannot serve as a definitive pathogenic indicator or clinical standard for determining the progression of MASLD.

Considering the protective role of TG discussed above, lipotoxicity is caused by harmful FFAs, especially NESFA, which are significantly elevated in obese patients with MASLD compared to obese patients with healthy livers.^86,87^ Notably, specific NESFA, such as stearic acid, increase proportionally with MASH severity, suggesting that, unlike monounsaturated fatty acids, they have pathogenic properties.^88^ FFAs function as inflammatory ligands by binding to specific receptors and activating intracellular signaling pathways that lead to macrophage activation and hepatocyte cell death. In addition, incorporation of NESFA into biological membranes, especially the ER, disrupts organelle function, exacerbating oxidative and ER stress and rendering hepatocytes more susceptible to cell death and inflammation. One hallmark of this inflammatory response is the formation of hepatic crown-like structures (hCLS), distinct liver lesions in which macrophages encircle lipid-laden hepatocytes. These structures are thought to be precursors to liver injury.^89^ Consistently, the presence of hCLS is significantly more frequent in the livers of patients with MASH compared to those with simple steatosis. Furthermore, the frequency of hCLS correlates positively with the severity of steatosis, hepatocellular ballooning, and liver fibrosis.^89^

There is ample evidence for the presence of ER stress in the MASH-afflicted liver, which is not detected at the benign MASL stage. Of note, ER stress is increased in proportion to the degree of liver fibrosis.^90^ In addition, ER stress markers were significantly upregulated in MASH-afflicted liver compared with livers from patients with MASL,^91^ implying a significant role in MASL to MASH progression.

### B. Endoplasmic reticulum stress is a significant contributor to metabolic dysfunction-associated steatohepatitis-related hepatocellular carcinoma onset

#### 1. IRE1 is a major regulator of hepatic lipotoxicity

In trying to understand how hepatocyte intrinsic ER stress promotes lipotoxicity, we employed high-fat diet (HFD)-fed *MUP-uPA* mice, a murine MASH and MASH-related HCC model characterized by hepatic ER stress and liver injury-associated inflammation (see below). In response to HFD feeding, the livers of *MUP-uPA* mice, but not WT controls, show activation of the lipogenic transcription factors sterol regulatory element binding protein 1 and 2 (SREBP1/2), which induce DNL and cholesterol biosynthesis.^28,91^ Given that the livers of HFD-fed WT mice exhibit extensive steatosis without inflammation and hepatic ER stress, we postulated that SREBP activation is linked to specific *MUP-uPA*-affiliated characteristics, like ER stress and inflammation. Moreover, persistent SREBP activation in the *MUP-uPA* liver stands in marked contrast to the cholesterol and FFA-mediated negative feedback control of SREBP activation mediated by the SREBP-cleavage activating protein (SCAP) and its negative regulator, insulin-induced gene.^92^ Indeed, examination of the molecular basis for SREBP1/2 activation in *MUP-uPA* mice revealed that the combination of ER stress and liver injury led to IRE1-dependent activation of a noncanonical pathway in which Casp2 constitutively activates the SREBP-activating S1P in the ER instead of the Golgi apparatus.^91^ Importantly, the activation of S1P by Casp2 does not require SCAP and is therefore not subject to feedback inhibition.^91,93^ Casp2 activation itself depends on assembly of a protein complex called the PIDDosome, which, in addition to Casp2, contains 2 other proteins: p53-induced protein with death domain and RIP-associated ICH-1/CED-3-interacting protein with death domain.^93^ Casp2 expression is induced upon IRE1 activation, thus explaining an earlier observation of ER stress-induced Casp2 activation in a manner dependent on IRE1.^43^ Consistent with these findings, an inhibitor of the IRE1 endoribonuclease, MKC3946, blocked hepatic lipid synthesis and MASL to MASH progression in HFD-fed *MUP-uPA* mice,^91^ as well as in high fructose diet (HFrD)-fed WT mice, in which fructose induces strong ER stress and Casp2-mediated SREBP activation.^93^ However, the exact role of Casp2-dependent SREBP1/2 activation in MASH development remains enigmatic and complex. Although we initially suggested that IRE1-dependent Casp2 activation promoted MASH development by upregulating hepatic DNL,^91^ our more recent results suggest that the main driver is the inhibition of canonical SREBP1/2 activation by its negative regulator insulin-induced gene-2, whose expression is IRE1 and c/EBP*β* inducible.^93^ Accordingly, we postulated that the lipids synthesized in response to noncanonical Casp2-dependent SREBP activation are more pathogenic than the protective, membrane-expanding lipids synthesized in response to canonical SREBP activation. Consistent with this hypothesis, Casp2 inhibition or ablation protected *MUP-uPA* mice from ER stress and MASH development.^91^ Importantly, Casp2 is also upregulated in MASH-bearing human livers compared with healthy or MAFL-affected livers, coinciding with ER stress and elevated expression of enzymes that support hepatic cholesterol biosynthesis.^91^ Curiously, S1P, which is activated by Casp2, is also known to cleave and activate ATF6, thereby promoting the expression of the important ER chaperone Grp78/BiP.^50,53,57^

The IRE1 substrate, XBP1, was also implicated in hepatic lipotoxicity and MASH pathogenesis.^94,95^ A study using hepatocyte-specific XBP1 knockout (KO) mice revealed spontaneous hepatic lipid accumulation but reduced circulating triglycerides and cholesterol, indicative of impaired lipid translocation and intracellular trafficking. In line with this, *Xbp1*-deficient livers displayed reduced expression of genes encoding enzymes critical for intracellular lipid transportation. Although hepatic XBP1 does not directly regulate the SREBP pathway, liver-specific XBP1 KO mice exhibit a marked reduction in hepatic lipogenesis, indicating that the XBP1 pathway is functionally interconnected with lipid biosynthetic processes.^96^ Further evidence from Wang et al^97^ demonstrated that the IRE1-XBP1 axis regulates expression of protein disulfide isomerase (PDI) and microsomal triglyceride transfer protein, both of which are essential for hepatic very-low-density lipoprotein assembly and secretion.

Notably, although XBP1-driven metabolic alterations can occur independently of dietary stimuli or overt metabolic stress,^96^ Casp2-mediated SREBP activation does not take place under basal conditions while becoming active upon hepatic injury, such as in HFD or HFrD-fed *MUP-uPA* mice.^91^ By governing 2 distinct molecular branches, IRE1 signaling serves as a metabolic rheostat that fine-tunes hepatic lipid metabolism in response to various environmental cues.

#### 2. IRE1-regulated cell death

Beyond signaling through cell-surface receptors, FFAs provoke ER stress and activate the UPR by altering membrane structure and physical properties,^70^ thereby contributing to MASH-related HCC progression. A central effector in this process is c-Jun N-terminal kinase (JNK), a stress-inducible Ser/Thr kinase activated by MAP3K-MAP2K signaling cascades.^98^ Upon activation by extracellular or intracellular cues, MAP3Ks phosphorylate MAP2Ks, which in turn phosphorylate JNK at the conserved Thr-Pro-Tyr motif.^98,99^ Activated JNK orchestrates a plethora of cellular responses, including apoptosis, proliferation, and reactive oxygen species (ROS) metabolism.^98,100^ Mechanistically, JNK regulates transcription via activator protein-1 (AP-1) family transcription factors while simultaneously modulating cytoplasmic effector proteins.^101^ To date, over 50 JNK substrates have been identified, including key pro- and anti-apoptotic Bcl-2 family members such as Bcl-2-associated X protein, Bcl-2 antagonist/killer 1, Bcl-2, and Bcl-xL, which mediate mitochondrial pore formation and execute apoptosis.^101^

TNF is one of the best-characterized JNK activators.^12^ Upon binding of trimeric TNF to its type 1 receptor (TNFR1), the trimeric membrane-associated complex I is assembled, comprising TNFR1-associated death domain, receptor-interacting Ser/Thr protein kinase 1, TNF receptor—associated factor 2, and cellular inhibitors of apoptosis.^102,103^ This complex leads to MAP3K activation, initiating JNK phosphorylation.^12^ In addition to JNK, TNFR1 effector engagement leads to activation of the IκB kinase and transcription factor NF-κB, whose activation induces transcription of numerous genes that orchestrate host defense mechanisms and suppress cell death, ultimately promoting cell survival.^104^ Besides this, TNF link to cell survival, Urano et al^105^ demonstrated that IRE1 also leads to JNK activation through direct binding to TRAF2, which may promote cell death. The ability of IRE1 and TNF to trigger such dual responses underscores the need to better understand the consequences of UPR activation under different levels of cell stress. This issue resembles the pioneering results reported by Liu et al,^104^ according to which TNFR1 activation can result in either JNK-mediated cell death or NF-*κ*B-induced cell survival. Accordingly, inhibition of NF-*κ*B activation or PERK-regulated suppression of protein translation results in JNK-dominated cell death. Considering that NF-*κ*B activation is an immediate, but self-limiting, stress response mounted downstream to IRE1 activation,^106,107^ it appears that prolonged ER stress may lead to the JNK-initiated cell death program; the level and extent of metabolic stress should be tested to understand the outcomes of IRE1-TRAF2 signaling.

The IRE1-regulated Casp2 (IRE1-Casp2) pathway was initially proposed to mediate apoptosis via the canonical BAX/BAK-dependent mitochondrial death axis.^43^ Nonetheless, our findings revealed that Casp2-induced hepatocyte apoptosis may result from lipotoxicity-driven organellar dysfunction, rather than direct activation of the mitochondrial apoptotic machinery.^93^ Hepatocyte-specific ablation of SCAP, a key regulator of SREBP1/2 activation and lipid biosynthesis, significantly reduces intracellular lipid availability.^108^ When SCAP ablation was combined with hepatic PTEN loss, mice exhibited dramatically accelerated HCC development, presenting with multiple hepatic nodules by 7 months of age.^109,110^ As PTEN suppresses insulin signaling and oncogene expression,^111^ the enhanced liver injury observed in SCAP/PTEN double-knockout (DKO) mice likely stems from hepatocyte death rather than insulin resistance. Remarkably, reconstitution of SREBP1 activity in SCAP/PTEN DKO livers substantially ameliorated hepatic damage and ER stress, indicating that the failure to expand the ER membrane in stressed mice is a key factor underlying liver damage.

To further probe the role of lipid depletion in organellar stress and MASH aggravation, we challenged hepatocyte-specific SCAP KO mice with a fructose-enriched diet, a well established inducer of hepatic lipogenesis and inflammation.^112^ Unlike SCAP-flox controls, SCAP-ablated mice displayed severe hepatic injury, including pronounced inflammation and portal fibrosis, resembling the pathology of SCAP/PTEN DKO mice.^93^ Notably, SCAP-deficient livers showed marked activation of ER stress pathways, including IRE1-Casp2 signaling and its downstream target, S1P.^93^ In these mice, hepatocyte apoptosis was confirmed by robust caspase-3 (Casp3) activation, consistent with the proposed IRE1-Casp2-Casp3 apoptotic cascade.^43^ However, strikingly, reactivation of SREBP1 in SCAP-deficient livers restored lipid homeostasis, resolved ER stress, and attenuated both IRE1-Casp2 signaling and Casp3-dependent cell death. These data suggest that replenishment of ER membrane lipids mitigates ER stress and cell death, highlighting the central role of lipid imbalance and organelle dysfunction in hepatocyte death. Nonetheless, the precise mechanistic link between IRE1-Casp2 signaling and executioner Casp3 activation under lipid-deprived conditions remains to be fully elucidated.

#### 3. PERK-directed defense against oxidative stress and ferroptosis

The ER lumen provides a specialized oxidizing environment that enables disulfide bond formation, a process essential for protein maturation. This is mediated by a sophisticated folding machinery centered on PDIs, which catalyze thiol-disulfide oxidation, reduction, and isomerization. Efficient disulfide bond formation requires ERO1, a flavin adenine dinucleotide-dependent oxidoreductase that transfers electrons from reduced PDI to molecular oxygen, generating ROS as a byproduct. Consequently, PDI-mediated oxidative folding generates oxidative stress, with ER protein folding machinery estimated to account for nearly 25% of all cellular ROS production.^113^ Maintaining redox balance is therefore a fundamental requirement for sustaining ER folding capacity. Within the UPR, the PERK branch serves as the primary regulator of antioxidant defenses, largely through its control of nuclear factor erythroid 2-related factor 2 (NRF2), a master transcriptional regulator of redox homeostasis.^114^ PERK regulates NRF2 by 2 complementary mechanisms: PERK-ATF4 signaling induces transcription of the NRF2 encoding *Nfe2l2* gene,^115^ and PERK directly phosphorylates NRF2, promoting its stabilization and nuclear localization.^116^ Together, these mechanisms ensure robust activation of NRF2 target genes, thereby strengthening cellular antioxidant defenses. Functionally, PERK-NRF2 signaling confers broad cytoprotective effects, including resistance to ER stress-induced apoptosis,^116^ as well as enhanced survival under chemotherapy^117^ and radiotherapy.^118^ NRF2 was recently shown to play a key role in the development of MASH-related HCC, allowing DNA-damaged HCC progenitor cells to escape senescence and progress to cancer.^119^ NRF2 was also found to activate lipid-metabolizing genes and promote hepatomegaly.^120^

Iron deposition and lipid peroxidation are one of the clinical features of MASH, and iron-induced ferroptosis was implicated in the development of MASH-associated HCC.^121^ Excessive iron deposition catalyzes lipid peroxidation primarily by generating ROS, thereby driving ferroptotic cell death.^122^ Owing to easy redox cycling between its ferrous (Fe^2+^) and ferric (Fe^3+^) states, iron serves not only as a cofactor for cytochromes and oxygen-binding proteins but also as a potent generator of ROS via the Fenton reaction, in which hydrogen peroxide is converted into highly reactive hydroxyl radicals.^123^ Consequently, systemic and cellular iron homeostasis must be tightly regulated. Hepcidin, a hepatocyte-derived peptide hormone, is a central regulator of liver iron homeostasis^124^ and modulates plasma iron levels through interaction with ferroportin, the cellular iron-exporting protein.^123^ Once polyunsaturated fatty acids (PUFA) are converted to PUFA-membrane phospholipids, catalyzed by coenzyme A (CoA) and acyl-CoA synthetase long-chain family member 4.^125^ PUFA-Pls subsequently engage in peroxidation reactions catalyzed by lipoxygenase or cytochrome P450 oxidoreductase, generating lipid peroxides.^126^ Antioxidant systems that detoxify ROS are therefore critical for constraining ferroptosis and preventing inflammation secondary to hepatocyte death.^126^ In murine models, an iron-enriched diet markedly increases hepatic lipid peroxidation and ferroptosis, producing histological features characteristic of MASH. Moreover, pharmacological inhibition of ferroptosis in these mice ameliorated MASH pathology and attenuated disease progression.^127^

Recent work from our lab has shown that ATF4 is a critical inducer of SLC7A11, a key component of the cysteine-glutamate antitransporter.^128^ Hepatocyte-specific ATF4 deletion (Atf4^ΔHep^) in HFD-fed *MUP-uPA* mice did not alter baseline phenotypes, but under diet-induced obesity, *Atf4*^Δ*Hep*^ mice exhibited increased iron accumulation and exacerbated ferroptosis in their livers, with extensive fibrosis and other MASH markers.^128^ This observation aligns with prior evidence that the PERK-ATF4 pathway provides ferroptosis resistance.^129^ Importantly, phenotypes of *Atf4*^Δ*Hep*^ mice closely resemble those of hepatocyte-specific NRF2 KO (*Nrf2^ΔHep^*) mice. Mechanistically, PERK-ATF4 induces the cystine-glutamate antiporter SLC7A11, whose expression facilitates glutathione synthesis needed for antioxidant defenses and inhibition of ferroptosis.^120,128^ These findings underscore ER stress signaling as a critical modulator of lipotoxicity-driven hepatocyte death, macrophage recruitment, and inflammatory amplification, all of which are instrumental pathogenic features of MASH-related HCC.^105,130^ Conversely, pharmacological ferroptosis inducers, including glutathione peroxidase 4 inhibitors, glutathione synthesis inhibitors (buthionine sulfoximine), and Na^+^-independent, cysteine-glutamate X^−^_c_ antiporter (sorafenib and erastin), profoundly remodel the hepatic transcriptional landscapes, prominently upregulating PERK-ATF4 target genes such as *CHOP*, *ASNS*, and *FGF19.*^128^ This bidirectional interplay suggests that ferroptosis not only is regulated by ER stress pathways but also gives feedback to modulate ER stress signaling.

Importantly, other UPR components can also participate in iron regulation in hepatocytes. The ER stress-activated IRE1-XBP1 axis augmented hepcidin expression.^131^ Likewise, the ER-resident bZIP transcription factor cAMP-responsive element binding protein H, activated via regulated RIP, the same mechanism that activates ATF6 during ER stress, drives acute-phase responses, one of which is the induction of hepcidin by binding to consensus elements within the *HAMP* promoter. Consistently, cAMP-responsive element binding protein H deficiency abrogates ER stress-induced hepcidin expression, resulting in hepatic iron accumulation.^131–133^

Despite multiple reports linking ER stress to hepatocyte apoptosis and ferroptosis, it should be considered that necroptosis is only one of several forms of cell death that take place in the MASH-afflicted liver. RIP3 is a component of the necrosome that activates the necroptosis effector mixed lineage kinase domain-like pseudokinase, whose levels are increased in MASH-bearing livers.^134^ Although the pathophysiological significance of nec- roptotic hepatocyte death is not fully understood, studies suggest that hepatic necroptosis may be a potential target for treatment of MASH-associated HCC.^135^

#### 4. NESFA-mediated stress signaling in the liver

Although TG synthesis and deposition, along with cholesterol esters in lipid droplets, are protective, NESFA are toxic.^136^ Indeed, NESFA often serves as a pathogenic indicator in patients with MASLD, as NESFA and cholesterol synergistically trigger organellar stress that stimulates the progression of MAFL to MASH. NESFA induces ER stress in the context of MASLD.^87,88,137,138^ Increased NESFA levels within the ER membrane were found to be associated with upregulation of the ER chaperone Grp78/BiP and increased liver injury in patients with MASLD.^88,139^ Importantly, NESFA- induced ER stress and liver injury are not related to the degree of hepatosteatosis and may occur independent of obesity.^140^ Cu-mulative studies have also suggested that changes in NESFA amounts correlate with MASLD pathogenesis.^88^ Lipidomic analyses of liver biopsies, plasma, and urine obtained from patients with MAFL, MASH, and cirrhosis revealed that short-chain and saturated fatty acyl-containing lipid species are abundant, whereas PUFA, which have protective properties, are less preva- lent.^141^ Interestingly, DAG, a lipid intermediate suggested to have a prognostic value in type 2 diabetes (T2D), is significantly less abundant in the livers of fibrosis-affiliated MASH compared with those with MAFL.^88,141^ When circulating lipids were analyzed, it was found that inflammatory lipids generated via the lipoxygenase pathway, such as 5-hydroxyeicosatetraenoic acid (HETE), 8-HETE, and 15-HETE, were significantly upregulated in the plasma of patients with MASH compared to patients with MAFL or healthy individuals.^88^ In studies using mouse macrophages as a model, we have shown that NESFA, like palmitate and stearate, alter organ- ellar membrane properties and promote the formation of lipid rafts that support the clustering of receptors that ultimately lead to JNK activation and execution of apoptosis and inflammation, while the presence of palmitoleic acid prevented JNK activation and cell death.^70^

Consistent with these results and despite the absence of in-depth mechanistic studies, NESFA have been considered major mediators of hepatic lipotoxicity and cell death, a pathological feature that distinguishes MASH from MAFL.^95,134,142^ Intriguingly, prolonged exposure to NESFA induces UPR-directed hepatocyte death through the direct upregulation of PUMA, a p53-inducible BH3-only proapoptotic protein.^143^

## V. Metabolic dysfunction-associated steatohepatitis-related hepatocellular carcinoma animal models involving hepatic endoplasmic reticulum stress

### A. High-fat diet–induced metabolic dysfunction-associated steatotic liver models

It should be noted that HFD, in which 60% of the saturated fats come from lard, which is routinely used to induce obesity and MASL in mice, leads to hepatosteatosis but not to steatohepatitis.^144^ Given that genetically obese models such as *ob/ob* and *db/db* mice, despite their extreme obesity, develop only steatosis and insulin resistance, but fail to progress to MASH.^145^ It was suggested that although leptin deficiency predisposes to steatosis, HFD-induced leptin resistance paradoxically shields the liver from in-flammatory damage.^146,147^ Possibly, leptin resistance induced by HFD-induced obesity ironically prevents liver damage.^147^ In addition, HFD-fed livers rarely show overt ER stress. Of note, nitric oxide derived from HFD metabolism directly modifies IRE1 through S-nitrosylation, which suppresses its RNase activity regardless of phosphorylation status.^148^ This reveals a diet-specific regulatory layer that uncouples UPR signaling from protein misfolding burden.^148^ Above all, HFD-fed animals require additional ER stressors and inflammatory cues to convert lipid-laden hepatocytes into a lipotoxic state, thereby initiating a pathogenic cascade that drives MASH progression. Transcriptomic analysis of control and HFD-fed mice revealed upregulation of lipid and carbohydrate metabolism pathways and downregulation of amino acid catabolism, as well as genes related to cholesterol metabolism in the livers.^149^ Given that accumulation of free cholesterol into the intracellular organelle membranes and consequent cell death are prerequisites for liver damage and inflammation, which are closely linked to MASH progression and aggravation,^91,150^ it is implied that standard HFD requires cholesterol supplementation for MASH progression and aggravation to initiate a carcinogenic process.

### B. The endoplasmic reticulum stress-prone MUP-uPA mouse model

Studies using *MUP-uPA* mice, which overexpress a secreted protein, urokinase plasminogen activator (uPA), from the hepatocyte-specific major urinary protein (MUP) promoter,^151^ have provided strong experimental support for the role of hepatocyte ER stress in MASH-related HCC development.^28,76^ These transgenic mice overexpress uPA from the hepatocyte-specific MUP promoter, thereby exceeding the folding capacity of young hepatocytes.^28^ As a result, they experience ER stress and exhibit neonatal hemorrhaging and increased hepatocyte death, which gradually diminish by 2 months of age due to transgene extinction.^152^ These early events effectively prime *MUP-uPA* mice to ER stress induction when fed HFD or HFrD, which predisposes them to indolent inflammation.^28,76,93^

Sustained consumption of HFD or HFrD by *MUP-uPA* mice results in the development of MASH and its eventual progression to MASH-related HCC, which is not observed in WT BL6 mice on the same diets.^28,76^ MASH in *MUP-uPA* mice is characterized by hallmark features such as extensive steatosis, periportal and pericentral inflammation, hepatocellular injury, and extensive fibrosis.^28,91^ After 8 months of HFD or HFrD intake, *MUP-uPA* mice progress to typical MASH-related HCC.^28^

### C. Fructose as a dietary component prone to inducing endoplasmic reticulum stress

Fructose, the main ingredient of high fructose corn syrup (HFCS), is a lipogenic and inflammatory sugar.^153^ Of note, HFCS consumption has steadily increased since the 1960s, closely paralleling the rising incidence of metabolic diseases.^154,155^ During this period, the intake of saturated fat from red and processed meats remained relatively constant, suggesting that fructose is a major dietary contributor to the growing prevalence of metabolic diseases, including MASLD.^154^ Clinical studies strongly supported this postulation; 10 weeks of consuming a fructose-sweetened beverage providing 25% of daily caloric intake increased plasma uric acid and adipokines in obese subjects, with no comparable effect observed for the glucose-administered group.^156^ Similarly, a diet supplying 35% of energy from fructose for 7 days induced dyslipidemia and ectopic hepatic fat accumulation in healthy individuals.^156,157^ Moreover, chronic fructose consumption consistently provokes pronounced hepatic injury, characterized by increased inflammation, hepatocyte ballooning, and fibrosis, hallmark features of MASLD and MASH.^158^ In animal studies, rats fed HFrD, but not HFD or normal chow diet, developed precancerous hepatic lesions.^159^ Collectively, these findings indicate that fructose accelerates pathogenic pathways driving the progression of MASH and MASH-related HCC. Of particular concern is the rising consumption of fructose among adolescents, which may increase their long-term risk of metabolic disorders.^160^

One of the most pronounced physiological consequences of excessive fructose intake is robust hepatic DNL.^112^ When the ingested fructose load surpasses the intestine’s clearance capacity, the excess fructose spills over into the portal circulation and is delivered directly to the liver,^75^ where it undergoes rapid catabolism, driving lipogenesis more potently than glucose.^112^ Unlike glucose, whose glycolytic entry is regulated by hexokinase (HK) through phosphorylation at the C6 position to yield glucose-6-phosphate, fructose is metabolized via fructolysis, a pathway strictly dependent on ketohexokinase (KHK), which phosphorylates fructose at the C1 position to generate fructose-1-phosphate.^161^ Loss-of-function mutations in the predominant hepatic isoform, KHK-C, cause benign essential fructosuria, an autosomal recessive genetic disorder characterized by urinary fructose excretion due to impaired hepatic fructolysis.^162^ Consistently, KHK-deficient mice are protected from fructose-induced hepatic steatosis, even after 24 weeks of 30% fructose-containing drinking water.^163^ Despite the marked increase in hepatic DNL caused by fructose consumption, our studies with HFrD-fed *MUP-uPA* mice indicated that the major MASH-promoting effect of fructose was exerted in the intestine, where fructose metabolism results in barrier disruption.^76^ Fructose-induced barrier disruption results in low-grade endotoxemia and translocation of lipopolysaccharide and probably other inflammation-provoking bacterial metabolites via the portal vein into the liver. This inflammatory response results in increased production of TNF, which leads to the induction of *Casp2* transcription and eventual activation of the Casp2-dependent noncanonical SREBP signaling pathway.^76^ Importantly, transgenic expression of IL-6 signal transducer in intestinal epithelial cell results in barrier fortification^164^ and completely inhibits HFrD-induced MASH and HCC development in *MUP-uPA* mice.^76^ More recently, we found that IL-6 signal transducer expression in intestinal epithelial cell also protects mice from alcohol-induced liver disease.^165^ These studies highlight the importance of the gut-liver axis^166^ in the development of metabolic dysfunction-associated alcohol-related liver disease and MASH and underscore the importance of the gut microbiota in the control of liver metabolism. Accordingly, more emphasis should be placed on barrier fortification and microbiota-induced inflammation than on inhibition of hepatic DNL, which only accounts for about 25% of liver fat, as a novel interception for MASLD, MASH, and alcohol liver disease.

In addition to and above and beyond its barrier-disrupting activity, fructose is a potent activator of hepatic UPR. Mice fed fructose-rich diets exhibit robust activation of all 3 UPR sensors compared with starch-fed controls.^93,167^ Direct comparisons of glucose/starch versus fructose feeding revealed that fructose selectively triggered IRE1-Casp2-mediated SREBP activation and lipogenesis.^93^ Although these effects also depend on barrier disruption and endotoxemia, we found that pharmacological inhibition of IRE1’s endoribonuclease activity also blocked fructose-driven SREBP1/2 activation and attenuated steatosis.^93^

### D. Other diet-induced metabolic dysfunction-associated steatohepatitis-related hepatocellular carcinoma models

Feeding C57BL/6 mice with a Western diet (WD), containing saturated fats, carbohydrates, and cholesterol (0.2%—2% of total weight), supplemented with sucrose- and fructose-enriched drinking water (WD-SW), for 6 months induced obesity, dyslipidemia, and elevated plasma liver enzymes. Histological examination revealed hallmark features of MASH, including a 2-fold increase in both macrovesicular and microvesicular steatosis, extensive inflammatory infiltration, hepatocyte ballooning, and stellate cell activation.^168,169^ Prolonged administration of fructose- or sucrose-supplemented WD (which is also referred to as fast food diet/FFD) also leads to HCC development but with substantially longer latency than in *MUP-uPA* mice. Mice harboring the *Alms1* mutation (*foz/foz* mice) are hyperphagic and develop spontaneous obesity accompanied by metabolic syndrome.^170^ Within 12 weeks of WD-SW feeding, *foz/foz* mice manifest MASH characterized by extensive steatosis, inflammation, and grade 2—3 fibrosis (F2—F3), progressing to visible tumors and cirrhosis in nontumor regions by 24 weeks.^170^ Transcriptomic profiling of WD-SW-fed *foz/foz* mice identified 254 dysregulated genes, including *Col1A1*, *Lgals3*, *Spp1*, and *Trem2*, which overlap with those expressed in human MASH and regulate intercellular communication and immune responses.^170^ Similarly, isogenic BL6/129 mice, generated by crossing C57BL/6J with 129S1/SvImj strain fed WD-SW for a year, demonstrated progressive MASH pathology. In these mice, also known as DIAMOND mice, steatosis peaked at grade 3 by 8 weeks, later declining while portal inflammation and fibrosis intensified by 52 weeks, producing a fibrosis pattern closely resembling that of human MASH.^171^ Notably, the gene expression profiles of WD-SW-fed livers were found to most closely mirror those of MASLD patients compared to other diet-induced models.^20^ The Amylin liver NASH model (AMLN), which is based on replacing a portion of the lard in HFD with transfats derived from hydrogenated vegetable oil and increasing the cholesterol content to 2%, leads to liver histological features like those seen in MASLD patients, including extensive steatosis, significant inflammation, and lobular fibrosis, along with insulin resistance and hyperglycemia, but no hepatocyte ballooning.^172,173^ Transcriptomic analysis further revealed that the AMLN liver group showed increased expression of gene sets involved in cell death, monocyte recruitment, and stellate cell activation.^173^ To further simulate modern sedentary lifestyles, the AMLN diet was combined with restricted physical activity, yielding the American lifestyle-induced obesity syndrome (ALIOS) model. When ALIOS mice were additionally provided HFCS, they gained 10% more body weight with concomitant increases in hepatic TG and plasma cholesterol by 16 weeks.^174^ Remarkably, after a year or longer on the ALIOS diet, ~50% of B6/129 mice developed neoplastic lesions marked by HCC- associated genes, including *Sox9* and *Ctnnb1* (*β*-catenin).^175^

The MCD is a popular and quick model that generates MASLD histologic features, including liver steatosis, apoptosis, and inflammation, after 8 weeks of consumption. However, unlike the diets mentioned above, MCD leads to significant body weight loss and presents with hypoglycemia, which is inconsistent with the anthropometric parameters observed in MASLD patients.^4^ Of note, MCD-fed mice exhibited significant hepatic ER stress, which was alleviated by treatment with tauroursodeoxycholic acid, an ER stress inhibitor, leading to an improvement in liver steatosis and fibrosis.^176^ The choline-deficient, L-amino acid-defined diet, often used in combination with an HFD to prevent dramatic weight loss, and referred to as CDAHFD, induces MASH onset, as evidenced by significantly elevated histologic markers of MASH, including liver steatosis, inflammation, hepatocyte ballooning, and fibrosis, and pronounced hepatic ER stress after 12 weeks.^177,178^ After 24 weeks of feeding, this diet results in the development of MASH-associated HCC.^179^ However, the major limitation of this model lies in its induction of body weight (BW) loss. Because BW management represents one of the most effective therapeutic strategies for patients with early-stage MASLD, a diet-induced model that paradoxically reduces BW fails to faithfully capture the pathogenic mechanisms underlying obesity-driven MASLD progression.

## VI. Metabolic dysfunction-associated steatotic liver disease/metabolic dysfunction-associated steatohepatitis treatments and drug development

The main goal of MASLD management is to achieve clinical benefits, with a primary focus on improving liver-related outcomes, such as preventing cirrhosis decompensation, slowing liver function decline, and reducing mortality. Currently, in MASLD/MASH clinical trials, improvement is defined as the resolution of steatohepatitis without worsening fibrosis, as well as a regression of at least 1 stage in fibrosis without worsening steatohepatitis.^180^ Here, we summarize current candidates for MASH treatment, focusing on those whose actions are related to ER stress (Table 1). All of the mentioned clinical trials for these treatments were multicenter, randomized, double-blinded, placebo-controlled studies.

### A. Body weight management

Lifestyle-based BW reduction through caloric restriction and increased physical activity remains an effective therapeutic strategy for patients with early MASLD/MASH without advanced fibrosis. Behavioral BW loss programs, pharmacotherapy, and bariatric surgery, alone or in combination, can be employed to enhance weight reduction.^208^ A loss of ≥ 5% BW was found to be required for a meaningful histological improvement. In 1 study, ≥ 5% weight loss resolved steatohepatitis in 25% of patients, reduced MAS in 47%, and induced fibrosis regression in 19%.^181^ Greater weight loss yields superior outcomes: patients achieving >10% BW loss demonstrated MAS improvement in nearly all cases, with 45% showing fibrosis resolution.^181^ The magnitude of BW loss correlates directly with liver histological improvement, with each 1 kg reduction associated with a 0.83 U/L decline in ALT and a 0.77% decrease in hepatic steatosis.^209^ Sustaining BW loss, however, is challenging, and weight regain often reverses improvements in hepatic lipid content and stiffness. In a 39-month follow-up, only 30% of patients with initial ≥5% BW loss maintained their lower weight, whereas ~20% regained weight to baseline or higher.^183^ Interestingly, BW management appears to be equally effective in lean (BMI <25 kg/m^2^) and nonlean MASH (BMI ≥25 kg/m^2^) patients when comparable interventions, such as dietary modification and exercise, are applied. Although the absolute weight loss tends to be greater in nonlean individuals, a reduction of ≥5% in BW significantly improves key histologic features of MASH, including steatosis, inflammation, and hepatocyte ballooning, resulting in a lower MAS score.^182^

### B. Thyroid hormone agonists

Thyroid hormone (TH), particularly triiodothyronine (T3), is a central regulator of growth and metabolism.^210^ Excess TH release induces a hypermetabolic state with increased resting energy expenditure, weight loss, and improved cholesterol profiles, whereas hypothyroidism is linked to hepatosteatosis and heightened MASLD risk.^210^ TH biosynthesis depends on the ER-associated substructure, called an ER-Golgi intermediate compartment, where thyroxine (T4) is converted to T3 by type 2 deiodinase (D2).^210^ Mutations in D2 can cause its retention within the trans-Golgi network and induce ER stress, whereas pharmacologic inhibition of the UPR with 4-phenylbutyric acid restores D2-regulated T3 production and alleviates hypothyroid phenotypes in murine models.^211^

Basal metabolic rate (BMR) is a key determinant of TH-mediated metabolic control. Individuals with low energy expenditure are predisposed to long-term weight gain and obesity^212^ and circulating TH levels inversely correlate with BW, underscoring their roles in BMR regulation.^213^ Unlike classical ATP generation through oxidative phosphorylation, TH increases energy usage by driving cellular ionic gradients: TH stimulates Na^+^ influx and K^+^ efflux, enhancing both the activity and expression of Na^+^/K^+^ ATPase.^210,214^ TH also promotes Ca^2+^ leakage from the ER/sarcoplasmic reticulum into the cytoplasm, activating Ca^2+^/calmodulin-dependent pathways, whereas TH concurrently induces sarco/ER Ca^2+^-ATPase expression to restore Ca^2+^ homeostasis. This futile ionic cycling increases ATP turnover independent of mitochondrial respiration. TH also augments BMR through thermogenesis, a process that diverts mitochondrial electron flow away from ATP synthesis toward heat production. This pathway depends on uncoupling protein 1 (UCP1), which dissipates the proton gradient across the inner mitochondrial membrane.^215^ TH induces *UCP1* transcription by binding to cAMP response elements on its promoter and synergizes with norepinephrine to amplify UCP1 expression up to 20-fold in brown adipose tissue (BAT).^216^ Importantly, TH-regulated thermogenesis relies on the specificity of the 2 TH receptors (TRs): TR*α* or TR*β*. Although TR*β* predominantly drives UCP1 expression in BAT,^217^ TR*α* is critical for UCP1 induction during white adipose tissue browning.^218^

TH regulates hepatic cholesterol metabolism by interacting with retinoid X receptor *α* and binding to SREBP response elements to upregulate SREBP2, a master regulator of cholesterol biosynthesis. TH/ retinoid X receptor (RXR)-mediated SREBP2 activation induces low-density lipoprotein receptor expression^219^ but inhibits ATP binding cassette subfamily A member 1 (ABCA1), which mediates hepatic cholesterol efflux, by competing with liver X receptor (LXR) binding to the *ABCA1* promoter.^220^ Beyond cholesterol, TH influences lipogenesis through SREBP1-driven expression of enzymes such as acetyl-CoA carboxylase (ACC).^91^ ACC generates malonyl-CoA, which inhibits carnitine palmitoyltransferase 1 (CPT-1), thereby blocking lipid entry into *β*-oxidation and favoring lipogenesis.^221^ Mechanistically, TH directly enhances *ACC* transcription via cooperative binding of TR and SREBP1 to adjacent sites on the *ACC* promoter, stabilizing SREBP1 occupancy.^222^

In parallel, the hypothalamic-thyroid axis integrates systemic metabolic regulation.^223,224^ T3 signaling in the ventromedial hypothalamus activates AMP-activated protein kinase *α*, alleviating ceramide-induced ER stress in BAT and promoting thermogenesis. Concurrently, T3-AMPK-JNK signaling suppresses hepatic lipogenesis via vagal innervation.^223^ Thus, through coordinated hepatic and neuroendocrine pathways, TH enhances energy expenditure and mitigates steatosis.

Clinical studies show that subclinical hypothyroidism patients are at high risk of MASLD, and disease severity correlates with the degree of thyroid dysfunction, independent of metabolic syndrome.^225^ In mice, a TR*α* (P398H) mutation impairs peroxisome proliferator-activated receptor *α* (PPAR*α*) activation and downstream oxidative phosphorylation, leading to defective adipocyte lipolysis, visceral adiposity, and severe hepatic steatosis.^226^ To test TH’s therapeutic potential, MASLD mice fed WD plus 15% fructose water were treated with T3 (35 mg/kg) for 8 weeks. T3 markedly suppressed hepatic steatosis with reductions in TG, DAG, monoacylglycerol, and cholesterol, largely via enhanced autophagy.^227^ However, systemic T3 also promotes adipocyte lipolysis, increasing lipid flux to the liver and hepatic lipid load,^210^ which limits its therapeutic utility. To overcome this, liver-directed TR*β* agonists were developed. In MASLD models, these agonists robustly reduced steatosis, inflammation, and fibrosis by upregulating hepatic mitochondrial function and fatty acid oxidation.^210,228,229^ This study became the basis of TH-based therapeutics in MASLD.

Prior to the advent of specific TR*β* agonists, a synthetic T4 prohormone, levothyroxine, was clinically evaluated in patients with hepatic steatosis. In a randomized trial, MASLD patients with T2D received either placebo or levothyroxine, titrated to achieve serum TSH concentrations of 0.34—1.7 mIU/L over 16 weeks. Proton magnetic resonance spectroscopy revealed a significant ~12% reduction in intrahepatic lipid content in the levothyroxine group.^184^ Resmetirom, a liver-directed and *β*-selective THR agonist, has been tested for its therapeutic efficacy in MASH. In a phase 2 study, daily administration of 80 mg resmetirom for 36 weeks to patients with F1—F3 fibrosis resulted in MASH resolution without fibrosis worsening in 39% of treated patients, compared with placebo.^185^ More recently, a phase 3 trial tested 80 mg or 100 mg resmetirom for 58 weeks, showing MASH resolution without fibrosis worsening in 25.9% and 29.9% of the patients, respectively, whereas ≥1-stage fibrosis improvement without MASH worsening was observed in 24.2% and 25.9% of the patients, respectively.^186^ Based on these phase 3 outcomes, the U.S. Food and Drug Administration has recently approved resmetirom for the treatment of noncirrhotic MASH.

### C. Glucagon-like peptide 1 receptor agonists

Glucagon-like peptide-1 (GLP-1), secreted from intestinal L-cells, exerts coordinated metabolic control across the pancreas, liver, adipose tissue, and brain. A key mechanism centers on its ability to mitigate ER stress because chronic ER stress-driven *β*-cell failure is a central event in T2D.^230,231^ Pharmacological activation of GLP-1 signaling with the GLP-1 receptor agonist (GLP-1RA) exendin-4 markedly reduced islet ER stress in *db/db* mice, where ATF4 upregulated antioxidant gene expression, thereby protecting *β*-cells from ER stress-mediated apoptosis and preserving *β*-cell mass.^232^ In parallel, GLP-1-dependent activation of phosphatidy-linositol-3-kinase enhanced pancreatic and duodenal homeobox-1 DNA-binding activity, thereby promoting *β*-cell DNA synthesis and proliferative capacity.^232^ These cytoprotective and proliferative actions extend beyond the pancreas to peripheral metabolic organs. In diet-induced obese mice, another GLP-1 agonist, semaglutide, markedly reduced BW and hepatosteatosis through suppression of DNL and inhibition of the PERK-ATF4-CHOP arm of the UPR.^233^ Consistently, administration of the GLP-1 agonist liraglutide to *ob/ob* mice attenuated insulin resistance via dual mechanisms: inhibition of ER stress signaling (IRE1-XBP-1 and PERK-ATF4-CHOP) coupled with augmentation of AKT-dependent insulin signaling in adipose tissue.^230^ Beyond these direct cellular actions, GLP-1RAs also modulate systemic energy balance. By slowing gastric emptying and engaging hypothalamic circuits that regulate feeding behavior, GLP-1 reduces postprandial glycemic excursions and suppresses energy.^234–236^

With their capacity to improve insulin sensitivity, GLP-1As and GLP-1RAs have shown promising antiobesity and antidiabetic efficacy, extending to patients with MASH.^237^ In the LEAN trial, 52 patients were randomized to liraglutide or placebo, where 48 weeks of liraglutide treatment led to higher rates of MASH resolution without fibrosis worsening compared with placebo.^187^ A larger phase 2 trial in 320 patients with fibrosis stages F1—F3 demonstrated a dose-dependent benefit of semaglutide, with MASH resolution achieved in up to 59% of patients receiving 0.4 mg versus 17% in the placebo group. Although fibrosis improvement occurred in 43% of the 0.4-mg group, this was not significantly different from placebo.^188^ However, a subsequent study using once weekly 2.4 mg semaglutide showed no clear benefit on histologic resolution or fibrosis regression,^189^ despite consistent weight loss. Moreover, both studies reported gastrointestinal adverse effects, including nausea, constipation, and vomiting.^188 189^ The development of dual incretin agonists has extended these findings. In a phase 2 trial of 190 patients with F2—F3 fibrosis, tirzepatide (a combined GIP/GLP-1 receptor agonist) induced MASH resolution without fibrosis worsening in 44%—62% of patients, depending on dose, and fibrosis improvement in approximately half of treated individuals.^190^ Most recently, a phase 3 trial involving 1197 patients with stage F2—F3 fibrosis reported MASH resolution without fibrosis worsening in 62.9% of those receiving 2.4 mg semaglutide weekly versus 34.9% with placebo, whereas ≥1-stage fibrosis improvement was achieved in 36.8% of semaglutide-treated patients compared with 22.4% in placebo.^191^

### D. Peroxisome proliferator-activated receptors agonists

PPAR are nuclear receptors that are highly expressed in metabolic tissues, where they orchestrate lipid and glucose metabolism. Each of the 3 isoforms, PPAR*α*, PPAR*β/δ*, and PPAR*γ*, engages distinct transcriptional programs, but they are all capable of alleviating features of the metabolic syndrome.^238^ PPAR*α* agonists such as fibrates and omega-3 FAs lower TG, whereas PPAR*γ* agonists, including the glitazones, act as insulin sensitizers. Dual PPAR*α/δ* agonists, exemplified by elafibranor, combine triglyceride- and glucose-lowering effects with improved peripheral insulin action, underscoring the therapeutic breadth of PPAR modulation.

Beyond metabolic control, PPAR signaling intersects with ER stress pathways. For instance, PPAR*α*-deficient rats fed a fructose-enriched diet exhibited markedly elevated hepatic ER stress, evidenced by increased phosphorylation of eIF2*α* and upregulation of Grp78/BiP and Grp94, alongside pronounced hepatic lipid accumulation compared with PPAR*α*-sufficient counterparts.^239^ PPAR*α* deletion also markedly enhanced hepatic JNK activation, indicating a sustained stress response in these animals.^239^ Consistent with the protective role of PPAR*α* against UPR activation, administration of a PPAR*α/γ* dual agonist reduced serum TG and glucose levels by alleviating hepatic ER stress in *db/db* mice.^240^ In contrast, fenofibrate treatment in HFrD-fed WT mice increased ER stress markers but simultaneously inhibited lipogenesis, reduced steatosis, and improved insulin responsiveness.^241^ These paradoxical findings indicate that PPAR regulation of ER stress is highly context-dependent, shaped by metabolic state and cellular environment.

PPAR panagonists have been tested for efficacy in MASH resolution. Elafibranor, given daily at 80 or 120 mg for 52 weeks in patients with fibrosis stages F1—F3, reduced steatosis and inflammation but failed to achieve significant histological improvement.^192^ Lanifibranor, another pan-PPAR agonist, was evaluated in a phase 2 trial of patients with advanced fibrosis. After 24 weeks, 55% of the 1200 mg-received and 48% of the 800 mg-received group achieved a ≥2-point reduction in MAS without fibrosis worsening, compared with 33% of the placebo. Fibrosis improvement without MASH worsening occurred in 48% of the 1200 mg group, 34% of the 800 mg group, and 29% of placebo.^193^ Despite these histological benefits, treatment was limited by frequent adverse events, including gastrointestinal symptoms, edema, anemia, and occasional heart failure, and the trial did not meet its primary endpoint of MASH resolution.^193^

### E. Farnesoid X receptor activators

Farnesoid X receptor (FXR), a BA-activated nuclear receptor enriched in the liver and intestine, integrates cholesterol and BA metabolism.^242^ Primary BA, such as cholic acid (CA), chenodeoxycholic acid, deoxycholic acid, and lithocholic acid, act as high-affinity endogenous ligands bound to FXR. Upon activation, FXR heterodimerizes with RXR and binds FXR response elements to regulate transcription of genes controlling hepatic bile acid and xenobiotic metabolism.^243^ A key mechanism is the FXR/RXR-driven induction of intestinal FGF19, which acts in the liver to suppress bile acid biosynthesis, thereby establishing enterohepatic feedback regulation.^244^

Beyond FXR/RXR-mediated FGF19-regulated hepatic BA biosynthesis,^245^ FXR virtually controls all aspects of BA metabolism. The ER protein cytochrome p450 7A (CYP7A1) hydroxylates the 7*α*-position of cholesterol to produce 7*α*-hydroxylcholesterol, which produces various types of BA.^246^ In human hepatocytes, RXR*α*-bound LXR*α* (LXR*α*/RXR*α*) interacts with BA response element in the *CYP7A1* promoter and stimulates CYP7A1 expression.^247^ Interestingly, FXR acts as a negative competitor of this binding by using a small heterodimer partner 1 (SHP1), an atypical nuclear receptor that lacks its own DNA-binding domain and acts as a dominant repressor through protein-protein interaction.^248^ Once FXR is activated, it induces robust expression of SHP1, which restricts LXR*α* from binding to the *CYP7A1* promoter, resulting in the suppression of LXR*α*/RXR*α*-mediated CYP7A1 expression.^248,249^ CYP7A1-generated 7*α*-hydroxylcholesterol is hydroxylated by cytochrome p450 8B1 (CYP8B1) at the 12*α* position.^246^ The FXR-SHP1 axis similarly negatively regulates expression of CYP8B1, thereby inhibiting BA biosynthesis. In this reaction, SHP1 binds and sequestrates hepatocyte nuclear factor, a critical transcriptional activator of the *CYP8B1* gene.^250^ FXR also regulates intestinal BA absorption and translocation through transcriptional controls of proteins, such as apical sodium-dependent transporter,^251^ fatty acid-binding protein class 6, and organic solute transporter-*α* and *β*.^252^ In addition, FXR induces the expression of CYP3A family members that catalyze the hydroxylation of BA at different positions, thereby eliminating toxic substances in the liver.^253^ Moreover, FXR participates in hepatic drug metabolism by regulating uridine-5′-diphosphate-glucuronosyl transferase 2B4 and sulfotransferase 2A1.^253,254^

FXR expression is reduced in both MASLD and viral hepatitis,^255^ and its reconstitution in MASLD animals lowered serum TG and cholesterol and prevented hepatotoxicity.^256^ FXR-mediated metabolic advantages are exerted via diverse pathways. The FXR-SHP1 axis was suggested to suppress SREBP1c activation by capturing LXR*α*, an important transcriptional activator of SREBP1c, thereby decreasing hepatic lipogenesis.^257^ In parallel, FXR also represses the expression of *Scd-1, Dgat2*, and *Lpin1*. This, in turn, inhibits fatty acid absorption into hepatocytes, especially monounsaturated fatty acids and PUFA.^256^ Intestinal FXR activation promotes the browning of white adipose tissue and increases energy expenditure.^258^ Moreover, FXR activation was found to ameliorate ER stress-regulated hepatotoxicity. FXR also suppresses PERK-CHOP—mediated NLRP3 activation, thereby diminishing ER stress-induced inflammation.^255^ Moreover, FXR activation reduces ER stress markers and ameliorates lipotoxicity by decreasing toxic lipid species, such as ceramide and free cholesterol, while increasing cardiolipin levels in the WD-fed mouse liver.^259,260^

Obeticholic acid (OCA) is a synthetic BA analog that functions as a highly potent FXR agonist, with approximately 100-fold greater activity than chenodeoxycholic acid. Its therapeutic potential has been evaluated across several clinical trials in MASLD. Early proof of concept came from a small trial in MASLD patients with T2D, where once-daily OCA (25 or 50 mg) for 6 weeks improved insulin sensitivity in roughly 1-quarter of participants.^194^ Afterward, the FLINT trial tested OCA (25 mg) for 72 weeks in MASH patients, reporting histological improvement in ~45% of treated individuals compared with placebo, including significant resolution of steatohepatitis.^195^ Larger phases 2 and 3 trials further examined antifibrotic efficacy. In a phase 2 study of nearly 2000 patients with stage 1—3 fibrosis, interim analysis at 18 months revealed fibrosis regression (≥1 stage without MASH worsening) in 23% of those on 25 mg OCA, compared with 12% on placebo. Some patients also achieved MASH resolution without fibrosis worsening.^196^ The phase 3 REGENERATE trial confirmed that both 10 mg and 25 mg OCA significantly reduced fibrosis progression in MASLD patients (n = 1218). However, despite these histological benefits,^197^ the trial was halted because of high rates of adverse events, most notably dose-dependent pruritus.^197,261^ Combination approaches have also been explored. In 1 study, MASH patients received an FXR agonist (30 or 100 mg OCA) together with semaglutide (2.4 mg) for 24 weeks. This regimen improved hepatic steatosis and stiffness, but pruritus again remained a limiting side effect.^262^

### F. Fibroblast growth factor agonists

Fibroblast growth factors (FGFs) constitute a large family of 22 members, recognized for their diverse roles in embryonic development, tissue repair, and metabolic regulation.^263^ Unlike most FGFs, which bind tightly to FGF receptors (FGFRs) in a heparindependent manner, the FGF19 subfamily, including FGF19, FGF21, and FGF23, lacks a heparin-binding domain. This structural distinction reduces their affinity for local FGFRs and prevents them from acting mainly in a paracrine or autocrine fashion. Instead, they circulate throughout the bloodstream to exert endocrine effects, enabling the regulation of metabolic processes in organs far from their site of secretion.

#### 1. FGF19

Intestinal FGF19 is induced in the distal ileum upon fasting/refeeding and is secreted into the circulation, where it travels to the liver and binds to FGFR4. This enterohepatic signaling suppresses BA synthesis through CYP7A1 inhibition. Mechanistically, FGF19 induces hepatic expression of SHP1, which sequesters hepatocyte nuclear factor 1*α*, a transcriptional activator of CYP7A1, thereby downregulating CYP7A1 expression and limiting hepatic BA production.^248^

In addition, FGF19 enhances hepatic protein and glycogen synthesis through insulin-independent pathways.^264^ FGF19 was shown to activate 2 distinct molecular pathways that converge to enhance global protein synthesis. First, FGF19-mediated extracellular signal-regulated kinase (ERK) activation phosphorylates MAPK-interacting kinase 1, which subsequently phosphorylates Ser209 of eIF4E. Second, ERK activation phosphorylates the downstream kinase p90 ribosomal S6 kinase, which in turn phosphorylates ribosomal protein S6 and eIF4B. Activation of these translational regulators leads to an overall increase in protein synthesis, with the rate of hepatic protein production elevated by approximately 25%.^264^ By activating ERK- p90 ribosomal S6 kinase signaling, which phosphorylates glycogen synthase kinase 3*α* at Ser21 and GSK3*β* at Ser9, FGF19 inhibits GSK3 substrate, leading to dephosphorylation and activation of glycogen synthase (GS), thereby stimulating glycogen synthesis. Importantly, these effects occur independently of the canonical insulin-AKT-mTOR pathway. Although insulin rises rapidly within 1 hour of refeeding, FGF19 induction peaks later (~3 hours), suggesting that FGF19 coordinates the delayed and sustained phase of metabolic adaptation.^264^

Importantly, serum FGF19 levels are reduced in patients with MASLD compared with healthy controls.^265,266^ Restoring FGF19, or administering its analog, in a MASLD mouse model not only improved insulin sensitivity but also resolved histological features of the disease.^259^ Because FGF19 promotes protein synthesis, a process tightly linked to ER protein folding capacity, it is reasonable to suspect a mechanistic connection between FGF19 and ER stress. Indeed, intestinal ER stress has been shown to induce FGF19 expression through the PERK-ATF4 pathway, as the *FGF19* promoter contains an amino acid response element where ATF4 binds to enhance transcription of FGF19.^267^ Furthermore, FGF19 was reported to activate NRF2 signaling by inhibiting its degradative phosphorylation by GSK3*β* in HCC cells.^268^ Because NRF2 can partner with ATF4 to regulate expression of antioxidant genes in response to ER stress,^120,269^ FGF19 and ATF4 appear to act cooperatively. Together, they promote antioxidant defenses, limit ER stress-induced damage, and ultimately support cell survival under prolonged stress conditions.

#### 2. FGF21

Similar to FGF19, FGF21 binds to the FGFR1c-*β*-klotho complex and acts as a powerful regulator of metabolism. However, its actions are distinct from those of FGF19. FGF21 is mainly produced and secreted by the liver, and therefore, it is often referred to as a hepatokine. FGF21 has a wide range of beneficial metabolic effects. One of its key roles is lowering plasma TG.^270^ PPAR*α* is the major transcription factor that upregulates *FGF21* mRNA in the liver in response to starvation and ketogenic diet consumption.^271^ During fasting, FGF21 mobilizes lipids into the liver and adipose tissue, facilitating clearance of plasma TG and thereby supporting metabolic adaptation to nutrient stress.^271,272^ FGF21 increases pancreatic *β*-cell mass and insulin secretion, further enhancing peripheral insulin sensitivity.^273^ FGF21 also lowers plasma glucose, which improves overall glucose tolerance and insulin sensitivity. This effect is partly due to its ability to upregulate GLUT1 expression and suppress lipolysis in adipose tissue.^274,275^ Beyond glucose and lipid metabolism, FGF21 contributes to circadian rhythm control by influencing hormonal regulation and behavior.^276^ It also promotes energy expenditure in neonatal BAT; FGF21 induces thermogenic genes such as *Ucp1*, *Ppargc1a*, *Dio2*, and *Glut1*, enhancing heat production in response to milk intake.^277^ Similarly, in adults, FGF21 induces a thermogenic program and drives the “browning” of white adipose tissue through upregulation of PGC-1.^278,279^

Hepatic expression of FGF21 and its circulating levels are markedly elevated in patients with MASLD^280,281^ and in livers under carcinogenic stress.^282^ Notably, the amount of hepatic FGF21 correlates with the severity of liver steatosis,^283^ suggesting that FGF21 is closely linked to the disruption of lipid metabolism in MASLD.^283^ Several factors regulate hepatic FGF21 expression, with ER stress emerging as a central player. In animal studies, induction of ER stress using compounds such as tunicamycin, thapsigargin, or dithiothreitol led to a significant upregulation of hepatic FGF21. Mechanistically, the *FGF21* promoter contains an amino acid response element, to which ATF4 binds to enhance FGF21 expression^284,285^ and an ERSE, which interacts with XBP1 to drive IRE1-XBP1-dependent *FGF21* mRNA induction.^280^ Under conditions of high fructose intake, FGF21 expression is also activated by carbohydrate response element binding protein, a transcription factor that is rapidly upregulated in response to carbohydrate feeding.^286^ Although carbohydrate response element binding protein is activated shortly after fructose intake, IRE1-XBP1 signaling persists with prolonged fructose feeding; these pathways together suggest that FGF21 acts as a sustained defense mechanism against ER stress across different feeding states.

Whether FGF21 acts directly within the liver remains less clear. Normal hepatocytes constitutively express *β*-klotho but not FGFR1c.^287,288^ Although FGFR1c expression is upregulated in MASLD livers^289,290^ and in immortalized HepG2 cells,^289,290^ knockout of *β*-klotho in hepatocytes still preserves the systemic benefits of FGF21, such as reduced BW, decreased hepatic TG, and improved insulin sensitivity. These findings suggest that the intrinsic liver response to FGF21 may be dispensable and that its major metabolic benefits are mediated through other tissues. Indeed, beyond direct hepatic effects, FGF21 acts primarily on adipose tissue, where it increases glucose uptake and inhibits hormone-sensitive lipase, thereby reducing lipolysis. Such findings imply that improvements in adipose tissue metabolism then feed back to confer indirect protective effects on the liver.

According to these metabolic benefits, several clinical trials have evaluated FGF21 analogs as potential therapies for MASL and MASH. One such drug is pegozafermin, a long-acting glycopegylated FGF21 analog. In a trial of MASLD patients, participants received varying doses of pegozafermin or placebo for 16 weeks. The results showed that pegozafermin significantly reduced liver fat fraction compared with placebo.^198^ Another analog, efruxifermin, was tested in a study of 747 MASH patients with moderate fibrosis (stages F2 and F3). When patients were randomized to receive either efruxifermin or placebo, the proportion of patients who showed fibrosis improvement by at least 1 stage without worsening MAS was more than double in the efruxifermin group compared with placebo.^201^ Similarly, Loomba et al^202^ conducted a trial in 222 MASH patients with F2—F3 fibrosis, and participants received placebo or pegozafermin. Fibrosis improvement was observed in more than 20% of patients receiving pegozafermin, compared with only 7% in the placebo group. Moreover, over 23% of pegozafermin-treated patients improved their MASH resolution without worsening fibrosis, whereas this occurred in only 2% of the placebo group. Finally, another study demonstrated that efruxifermin treatment not only significantly reduced LFF but also led to at least a 1-stage improvement in fibrosis after 16 weeks, compared with placebo.^199^ Recent trials have also tested FGF21 analogs in patients with more advanced liver disease, fibrosis stages F3—F4, and compensated cirrhosis.^200,203,204^ In the FALCON1 study, 197 patients with MASH and F3 fibrosis were randomly assigned to receive either placebo or different doses of pegbelfermin for 48 weeks. The results showed that more than 30% of patients treated with pegbelfermin experienced a reduction in LFF. However, the outcomes for fibrosis regression and MASH resolution were not statistically significant.^203^ A similar trial, called FALCON2, tested pegbelfermin in patients with MASH and F4 fibrosis with compensated cirrhosis.^204^ The pegbelfermin group showed reductions in LFF and some improvements in inflammation, but the effects on fibrosis regression and MASH resolution did not reach statistical significance.^204^ Notably, a parallel trial tested efruxifermin in patients whose liver conditions were similar to those in FALCON2 (F4 fibrosis and compensated cirrhosis). The results were more encouraging: 33% of efruxifermin-treated patients showed at least a 1-stage improvement in fibrosis without worsening MASH, and an additional 25% of patients showed improvements in MASH resolution themselves. Importantly, efrux- ifermin treatment also reduced liver stiffness, a marker of tissue scarring.^200^ The adverse effects were detected in patients who received FGF21 analogs, such as mild-to-moderate diarrhea with and without nausea.

### G. Acetyl-CoA carboxylase and FA synthase inhibitors

ACC catalyzes the formation of malonyl-CoA, an essential lipid intermediate required for FA synthesis by FA synthase (FASN).^291^ ER stress activates SREBP, which in turn promotes the expression of ACC and stimulates DNL in MASLD.^91,93^ The ACC inhibitor, firsocostat (GS-0796), has been tested for its efficacy in resolving MASH in a phase 2 clinical trial; 126 patients with MASH-positive fibrosis were given 5 or 20 mg of firsocostat daily for 12 weeks. Using magnetic resonance imaging-proton density fat fraction to assess liver steatosis, the study found that ACC inhibition significantly reduced liver steatosis, with a 30% reduction in magnetic resonance imaging-proton density fat fraction in 47% of patients receiving 20 mg of firsocostat, compared with 15% in those given a placebo. However, the treatment was associated with serious adverse effects, including hypertriglyceridemia, which led to treatment discontinuation in 16 patients due to plasma TG exceeding 500 mg/dL.^205,292^ To address this issue, combination therapy was tested in which 22 patients with MASH received 2.4 mg of semaglutide and 20 mg of firsocostat for 24 weeks. This combination significantly resolved hyperglycemia, an adverse effect associated with ACC inhibition, but was still accompanied by sustained nausea, diarrhea, constipation, and reduced appetite.^205^ In another phase 2 clinical trial, an ACC inhibitor was combined with a DGAT2 inhibitor and administered to MASH patients. The combination of 10 mg of ACC inhibitor with 300 mg of DGAT2 inhibitor twice daily for 48 weeks resulted in a significant reduction in hepatosteatosis in 80% of patients. However, this combination also caused a dramatic upregulation of serum TG, reaching levels of 800 mg/dL, leading to a serious adverse effect that resulted in study discontinuation.^293^

The FASN inhibitor TVB-2640 was tested for its efficacy in resolving MASH in a trial with 19 patients, who received either 25 mg or 50 mg once daily for 12 weeks. The 50 mg dose of TVB-2640 significantly reduced LFF, with a 28% reduction rate, compared with just 4.5% in the placebo group.^206^ Another FASN inhibitor, denifanstat, was administered to 168 patients diagnosed with biopsy-confirmed MASH and fibrosis stages 2 or 3, once per day for 52 weeks. This phase 2b trial showed that 38% of the denifanstat-treated group experienced at least a 2-point improvement in the MAS without worsening fibrosis, compared with 16% in the placebo group. Notably, the most common adverse events were an increased risk of COVID-19, dry eye symptoms, and alopecia.^207^

### H. Summary

Altogether, recent clinical trials have advanced the treatment of patients with MASH and opened a new era of therapeutic possibilities; however, several limitations remain and require further refinements. One major challenge would be the assessment of histological fibrosis, which still relies on liver biopsy. This method has technical limitations, including significant interobserver and intraobserver variability.^190,294^ Unfortunately, it remains challenging to accurately examine the histology of MASH patients, particularly features such as liver steatosis, inflammation, and hepatocyte ballooning.^294^ These difficulties make the current methods of histological evaluation suboptimal and often inconsistent. Therefore, there is an urgent need for technical improvements that can provide a more comprehensive assessment of overall liver status and thereby enhance the accuracy of disease evaluation.^295^ Recent advances in artificial intelligence (AI) and machine learning have shown considerable promise in augmenting histological assessment and enhancing diagnostic accuracy. The AI-based measurement tool for MASH histology has demonstrated expert-level performance in the interpretation of digitized liver biopsy slides and the prediction of histological scores.^296,297^ In a reanalysis of histological specimens from a phase 2 clinical trial (NCT02970942), the AI-powered PathAI platform achieved high reproducibility in the identification of key histological features, yielding results concordant with those obtained through traditional pathological evaluation.^298^ These findings underscore the potential of AI-based platforms to facilitate more standardized, scalable, and reproducible assessment of MASH in both clinical and research settings.

Nevertheless, there are currently no well established candidate pipelines for investigating progression to MASH-related HCC. This gap largely stems from the limited number of translational studies conducted in appropriate experimental systems. A major barrier is the lack of animal models that faithfully recapitulate the molecular mechanisms driving the transition from MASH to HCC. One of the principal technical challenges is the difficulty of establishing lowgrade, chronic inflammation in animal systems, as defense capacity and immune system architecture differ substantially between species, and such differences may obscure the heterogeneity of pathogenesis observed in patients. The *MUP-uPA* model provides an important exception. In this system, transgene expression induces early-life liver injury, which primes the liver to remain highly sensitive to immune insults derived from high-calorie diets. This results in long-lasting, low-grade inflammation that more closely mirrors the human disease course. To advance understanding of MASH to HCC progression, it is likely that additional models with *MUP-uPA*-like features will need to be developed and systematically tested.

Another complementary approach would lie in integrating multiple advanced data sources, including hospital-based clinical studies, computational analyses of patient-derived biopsy data, and recent advances in biological and technical instrumentation. Moreover, the incorporation of AI could greatly enhance these efforts, enabling the design of experimental models that better reflect the diversity of pathogenic trajectories in patients with MASH-related HCC.

## VII. Conclusions and future prospects

Managing ER stress is indispensable for maintaining normal liver function. Tremendous progress has been made in elucidating the UPR and its role in MASLD, advancing our understanding not only of UPR mechanisms per se but also of their integration into diverse cellular and pathophysiological processes. The ER, with its phospholipid-based membrane providing elasticity, fluidity, and electrostatic convergence,^299^ forms a vast intracellular network that continuously interacts with other organelles. Among these, ER-mitochondria contacts are of particular relevance: both their frequency and extent correlate with fatty liver severity in MASLD patients^300^ and contribute to organellar dysfunction that predisposes hepatocytes to cell death.^301^

Genetic variation adds another layer of complexity. Variants in MASLD livers are closely associated with progression to MASH and HCC.^74^ Somatic mutations, often propagated under nutrient overload, have been postulated to promote hepatocellular lipid accumulation. Experimental evidence suggests that these mutations expand in the context of high-fat feeding, thereby exacerbating lipid deposition,^302,303^ although the precise molecular mechanisms remain unclear. Furthermore, the ER’s role as a continuum with the nuclear membrane implicates it in genomic instability: ER-derived nuclear structures contribute to micronucleus formation, whose reintegration or independence can significantly impact genome function.^304^ Collectively, these dynamic ER functions could potentially be instrumental for the progression from MASH to cirrhosis and/or HCC.

Recent clinical approvals of GLP-1 receptor agonists and TR*β* activators for MASH highlight the therapeutic importance of systemic regulation of liver function, particularly at the interface of the central nervous system (CNS) and peripheral tissues.^305^ Intriguingly, studies demonstrate that metabolic stress and CNS perception of food can directly innervate hepatocytes and activate insulin-mimetic signaling pathways that are distinct from canonical glucose-insulin regulation.^305,306^ Importantly, ER stress, particularly via IRE1 activation, emerged as a central mediator of this CNS-liver communication, regulating hepatic lipid and glucose metabolism in response to neural cues. These findings suggest that ER stress can be triggered not only by metabolic intermediates but also by neuronal inputs, revealing a new dimension of ER function in hepatocytes.

Taken together, MASLD is best understood as a chronic systemic disorder involving intricate multiorgan interactions. ER stress, operating both in peripheral tissues and the brain, acts as a key coordinator of whole-body metabolic homeostasis. Thus, targeting ER stress represents a promising therapeutic approach for the prevention and treatment of MASLD, a disease whose incidence is tightly coupled to maladaptive responses to a calorie-rich environment. Furthermore, future research should prioritize exploring the interconnected network of ER stress responses across organs, providing a holistic framework to understand ER-mediated metabolic outcomes and guide the development of novel therapeutic strategies.

## Figures and Tables

**Table 1:** Drug now currently testing for patients with MASL, MASH ± cirrhosis

Target	Year	Phase	Population	Fibrosis	Cirrhosis	Intervention	Dosing	Follow-up	Evaluation	Outcomes of Test	References
BW loss	2015	N/R	MASL (n = 293)	N/R	N/R	Low-calories diet	750 Kcal/d	52 wk	M/I M/R F/R	M/I (47%) M/R (25%) F/R (19%)	Vilar-Gomez et al^181^
BW loss	2019	N/R	Lean and Nonlean MASH (n = 40)	N/R	N/R	Diet restriction + exercise	Diet recommendation	1 y	M/I	>5% M/I found in nonlean, lean group	Alam et al^182^
BW loss	2022	N/R	Obesity or T2D	N/R	N/R	Questionnaire survey + dietary recommend	N/R	60 wk	BW regain	21.1% regained BW	Malespin et al^183^
THR-*β*	2018	Phase2	MASL +T2D (n = 20)	N/R	No	Levothyroxine	Titrated does to attain TSH level between 0.34 and 1.7 mLU/L	16 wk	LFF	Sig. reduction in LFF, improved HbA1C (75% to Lev. group)	Bruinstroop et al^184^
THR-*β*	2019	Phase 2	MASH (n = 348)	F1-F3	No	Resmetirom	80 mg Once daily	36 wk	M/R	M/R (37.7% to Res. 8.5% to placebo)	Harrison et al^185^
THR-*β*	2024	Phase 3	MASH (n = 966)	F1-F4	Yes	Resmetirom	80 mg or 100 mg Once daily	52 wk	M/R F/R	M/R (25.9% of 80 mg, 29.9% of 100 mg 9.7% to placebo) F/R (24.2% of 80 mg, 25.9% of 100 mg 14.2% to placebo)	Harrison et al^186^
GLP-1	2016	Phase 2	MASH (n = 52)	N/R	N/R	liraglutide	1.8 mg Once daily	48 wk	M/R	M/R (9% to Lig. 2% to placebo)	Armstrong et al^187^
GLP-1	2021	Phase 2	MASH (n = 320)	F1-F3	N/R	Semaglutide	0.1 mg 0.2 mg 0.4 mg Once daily	72 wk	M/R F/R	M/R (40% of 0.1 mg, 36% of 0.2 mg, 59% of 0.4 mg 17% to placebo) F/R (43% of 0.4 mg 33% to placebo)	Newsome et al^188^
GLP-1	2023	Phase 2	MASH	F1-F4	N/R	Semaglutide	2.4 mg Once weekly	48 wk	M/R F/R	No significant MASH resolution and fibrosis improvement	Loomba et al^189^
GLP-1 +GIP	2024	Phase 2	MASH (n = 190)	F2-F3	N/R	Tirzepatide	5 mg 10 mg 15 mg Once weekly	52 wk	M/R F/R	M/R (44% to 5 mg, 56% to 10 mg, 65% to 15 mg 10% to placebo) F/R (15% to 5 mg, 51% to 10 mg, 15 mg 30% to placebo)	Loomba et al^190^
GLP-1	2025	Phase 3	MASH (n = 1197)	F2-F3	N/R	Semaglutide	2.4 mg Once weekly	240 wk	M/R F/R	M/R (62.9% to Sem., 34.9% to placebo) F/R (36.8% to Sem., 22.4% to placebo)	Sanyal et al^191^
PPARs	2016	Phase 2b	MASH (n = 276)	F1-F3	No	Elafibranor	80 mg 120 mg Once daily	52 wk	M/R	No significant improvement was found	Ratziu et al^192^
PPARs	2021	Phase 2b	MASH (n = 247)	F2-F4	No	Lanifibranor	800 mg, 1200 mg Once daily	24 wk	M/R F/R	M/R (55% to 1200 mg, 33% to placebo) F/R (35% to 1200 mg, 9% to placebo)	Francque et al^193^
FXR	2013	N/R	MASLD + T2D (n = 64)	N/R	N/R	Obeticholic acid	25 mg, 50 mg Once daily	6 wk	Insulin sensitizing effect	Improved insulin sensitivity (28% to 25mg, 20% to 50 mg 5.5% to placebo)	Mudaliar et al^194^
FXR	2015	Phase 2	MASH (n = 283)	N/R	No	Obeticholic acid	25 mg Once daily	72 wk	M/R (at least 2 points)	M/R (45% to OCA, 29% to placebo)	Neuschwander-Tetri et al^195^
FXR	2019	Phase 2	MASH (n = 1968)	F1-F3	No	Obeticholic acid	10 mg, 25 mg Once daily	18 mo	M/R F/R	M/R (11% to 10 mg, 12% to 25 mg, 8% to Plac.) F/R (18% to 10 mg, 23% to 25 mg, 12% to placebo)	Younossi et al^196^
FXR	2022	Phase 3	MASH (n = 1218)	F1-F3	No	Obeticholic acid	10 mg, 25 mg Once daily	6,12,or 18 mo	F/R Adverse effect	OCA group showed F/R Adverse effect, (pruritus) Study was terminated	Younossi et al^197^
FGF21	2019	Phase 2a	MASH (n = 75)	N/R	N/R	Pegbelfermin	10 mg Once daily 20 mg Once weekly	16 wk	LFF	LFF reduction (6.88% to 10 mg, 5.2% to 20 mg, 1.3% to placebo)	Sanyal et al^198^
FGF21	2021	Phase 2a	MASH (n = 80)	F1-F3	N/R	Efruxifermin	28 mg 50 mg 70 mg Once weekly	16 wk	LFF	LFF reduction (More than 12% reduction in all group 0.3% to placebo)	Harrison et al^199^
FGF21	2023	Phase 2a	MASH (n = 30)	F4	Yes	Efruxifermin	50 mg Once weekly	16 wk	Safety tolerance test	Intervention appeared to be safe and tolerant	Harrison et al^200^
FGF21	2023	Phase 2b	MASH (n = 747)	F2-F3	N/R	Efruxifermin	28 mg 50 mg Once weekly	96 wk	F/R	By 24 wk F/R (39% to 28 mg, 41% to 50 mg, 20% to placebo)	Harrison et al^201^
FGF21	2023	Phase 2b	MASH (n = 222)	F2-F3	N/R	Pegozafermin	15 mg 30 mg Once weekly 44 mg Once biweekly	24 wk	M/R F/R	M/R (37% to15 mg, 23% to30 mg, 26% to 44 mg, 2% to placebo) F/R (22% to 15 mg, 26% to 30 mg, 27% to 44 mg, 7% to placebo)	Loomba et al^202^
FGF21	2024	Phase 2b	MASH (n = 197)	F3	N/R	Pegbelfermin	10 mg 20 mg 40 mg Once weekly	48 wk	M/R	By 48 wk, fibrosis improvement and MASH resolution were detected but no statistical significance	Loomba et al^203^
FGF21	2024	Phase 2b	MASH (n = 155)	F4	YES	Pegbelfermin	10 mg 20 mg 40 mg Once weekly	52 wk	M/R F/R	Primary end points were not reached	Abdelmalek et al^204^
ACC	2018	Phase 2	MASH (n = 126)	F1-F3	N/R	Firsocostat	5 mg 20 mg Once daily	12 wk	LFF	LFF reduction (30% to Fir., 15% to placebo)	Alkhouri et al^205^
FASN	2021	Phase 2a	MASH (n = 99)	N/R	N/R	TVB-2640	25 mg 50 mg Once daily	16 wk	LFF	LFF reduction (9.6% to 25 mg, 28.1% to 50 mg, 4.5% to placebo)	Loomba et al^206^
FASN	2024	Phase 2b	MASH (n = 108)	F1-F2	N/R	Denifanstat	50 mg Once daily	52 wk	M/R	M/R (38% to Den., 16% to placebo)	Loomba et al^207^

F/R, fibrosis regression at least ≥1 without MASH worsening; LFF, liver fat fraction; M/I, MAS improvement; M/R, MASH resolution without fibrosis worsening; mo, months; N/R, not reported; T2D, type 2 diabetes; wk, weeks.

## Data Availability

There are no datasets presented in this paper.

## Significance Statement:

The endoplasmic reticulum (ER) functions as a central signaling hub, transmitting stress cues to transcriptional and translational programs through activation of the unfolded protein response, which orchestrates adaptive responses required for stress recovery. Given that hepatocytes are the largest cell population responsible for systemic protein distribution through ER-regulated protein synthesis, precise control of hepatic ER stress is essential not only for maintaining normal hepatocyte function but also for developing therapeutic strategies against ER stress-driven metabolic dysfunction-associated steatotic liver disease.

## Abbreviations

ABCA1: ATP binding cassette subfamily A member 1
ACC: Acetyl-CoA carboxylase
AI: artificial intelligence
ALIOS: the American lifestyle-induced obesity syndrome
AMLN: the Amylin liver NASH model
AMPK: AMP-activated protein kinase
AP-1: activating protein-1
ATF4: activating transcription factor 4
ATF6: activating transcription factor 6
BA: bile acids
BAT: brown adipose tissue
BMI: body mass index
BMR: basal metabolic rate
bZIP: basic leucine zipper
Casp2: caspase2
Casp3: caspase3
CA: cholic acid
CDAA: choline-deficient, L-amino acid-defined
chREBP: carbohydrate response element binding protein
CNS: central nervous system
CPT-1: carnitine palmitoyltransferase 1
CYP7A1: cytochrome P450 7A
DAG: diacylglycerol
DNL: de novo lipogenesis
DGAT: diacylglycerol acyltransferase
D2: type 2 deiodinase
eIF2*α*: *α*-subunit of eukaryotic translation initiation factor 2
EM: electron microscopy
ERAD: ER-associated degradation
ERK: extracellular signal-regulated kinase
ERSE: ER stress-response element
FASN: FA synthase
FFA: free fatty acids
FGF: fibroblast growth factor
FGFR: FGF receptor
GLP-1: glucagon-like peptide-1
GLUT: glucose transporter
Grp78/BiP: 78 kDa glucose-regulated protein
GS: glycogen synthase
GSK3*α*: glycogen synthase kinase 3*α*
hCLS: hepatic crown-like structure
HCC: hepatocellular carcinoma
HETE: hydroxyeicosatetraenoic acid
HFD: high-fat diet
HFCS: high fructose corn syrup
HFrD: high fructose diet
HK: hexokinase
IL6: interleukin 6
IRE1: inositol-requiring enzyme 1
JNK: c-JUN N-terminal kinase
KHK: ketohexokinase
KO: knockout
LXR: liver X receptor
MAS: MASLD activity score
MASLD: Metabolic dysfunction-associated steatotic liver disease
MASH: Metabolic dysfunction-associated steatohepatitis
MASL: Metabolic dysfunction-associated steatotic liver
MCD: methionine-choline deficient diet
miRNA: microRNA
NESFA: non-esterified saturated fatty acids
NF-Y: nuclear factor Y
NRF2: erythroid 2-related factor 2
OCA: obeticholic acid
PDI: protein disulfide isomerase
PERK: protein kinase RNA-like ER kinase
PPAR: peroxisome proliferator-activated protein
PUFA: polyunsaturated fatty acids
RER: rough ER
RIDD: regulated IRE1-dependent mRNA decay
RIP: regulated intramembrane proteolysis
ROS: reactive oxygen species
RXR: retinoid X receptor
SCAP: SREBP-cleavage activating protein
SER: smooth ER
SHP1: small heterodimer partner
SREBP: sterol regulatory element-binding protein
S1P: site-1 protease
TG: triglyceride
TH: thyroid hormone
TNF: tumor necrosis factor
TNFR1: TNF receptor type 1
TRAF2: TNFR-associated factor 2
TRs: TH receptors
T2D: type 2 diabetes
T3: triiodothyronine
T4: thyroxine
UCP1: uncoupling protein 1
UPR: unfolded protein response
uPA: urokinase plasminogen activator
WD: western diet
XBP1: X-box binding protein 1

## References

[1] Mandon EC, Trueman SF, Gilmore R. Protein translocation across the rough endoplasmic reticulum. Cold Spring Harb Perspect Biol. 2013;5(2):a01334. 10.1101/cshperspect.a013342 PMC3552503 23251026

[2] Schroder M, Kaufman RJ. ER stress and the unfolded protein response. Mutat Res. 2005;569(1–2):29–63. 10.1016/j.mrfmmm.2004.06.056 15603751

[3] Braakman I, Hebert DN. Protein folding in the endoplasmic reticulum. Cold Spring Harb Perspect Biol. 2013;5(5):a013201. 10.1101/cshperspect.a013201 23637286 PMC3632058

[4] Karin M, Kim JY. MASH as an emerging cause of hepatocellular carcinoma: current knowledge and future perspectives. Mol Oncol. 2024;19(2):275–294. 10.1002/1878-0261.13685 38874196 PMC11793012

[5] Vembar SS, Brodsky JL. One step at a time: endoplasmic reticulum-associated degradation. Nat Rev Mol Cell Biol. 2008;9(12):944–957. 10.1038/nrm2546 19002207 PMC2654601

[6] Walter P, David R. The unfolded protein response: from stress pathway to homeostatic regulation. Science. 2011;334:1081–1085. 22116877 10.1126/science.1209038

[7] Cohen JC, Horton JD, Hobbs HH. Human fatty liver disease: old questions and new insights. Science. 2011;332:1519–1523. 21700865 10.1126/science.1204265 PMC3229276

[8] Rinella ME, Lazarus JV, Ratziu V, A multisociety Delphi consensus statement on new fatty liver disease nomenclature. Hepatology. 2023;78(6):1966–1986. 10.1097/HEP.0000000000000520 37363821 PMC10653297

[9] Pais R, Barritt ASt, Calmus Y, NAFLD and liver transplantation: current burden and expected challenges. J Hepatol. 2016;65(6):1245–1257. 10.1016/j.jhep.2016.07.033 27486010 PMC5326676

[10] Danpanichkul P, Suparan K, Kaeosri C, Global trend of MASH-associated liver cancer: a systematic analysis from the global burden of disease 2021. Clin Gastroenterol Hepatol. 2025;23(8):1346–1355. 10.1016/j.cgh.2024.10.026 39694213 PMC12167746

[11] Villanueva A Hepatocellular Carcinoma. N Engl J Med. 2019;380(15):1450–1462. 10.1056/NEJMra1713263 30970190

[12] Sun B, Karin M. Obesity, inflammation, and liver cancer. J Hepatol. 2012;56(3):704–713. 10.1016/j.jhep.2011.09.020 22120206 PMC3889660

[13] Suresh D, Srinivas AN, Kumar DP. Etiology of hepatocellular carcinoma: special focus on fatty liver disease. Front Oncol. 2020;10:601710. 10.3389/fonc.2020.601710 33330100 PMC7734960

[14] George ES, Sood S, Broughton A, The association between diet and hepatocellular carcinoma: a systematic review. Nutrients. 2021;13(1):172. 10.3390/nu13010172 33430001 PMC7826815

[15] Miao L, Targher G, Byrne CD, Cao YY, Zheng MH. Current status and future trends of the global burden of MASLD. Trends Endocrinol Metab. 2024;35(8):697–707. 10.1016/j.tem.2024.02.007 38429161

[16] Llovet JM, Willoughby CE, Singal AG, Nonalcoholic steatohepatitis-related hepatocellular carcinoma: pathogenesis and treatment. Nat Rev Gastroenterol Hepatol. 2023;20(8):487–503. 10.1038/s41575-023-00754-7 36932227 PMC12165718

[17] Angulo P, Kleiner DE, Dam-Larsen S, Liver fibrosis, but no other histologic features, is associated with long-term outcomes of patients with nonalcoholic fatty liver disease. Gastroenterology. 2015;149(2):389–397.e10. 10.1053/j.gastro.2015.04.043 25935633 PMC4516664

[18] Ekstedt M, Hagstrom H, Nasr P, Fibrosis stage is the strongest predictor for disease-specific mortality in NAFLD after up to 33 years of follow-up. Hepatology. 2015;61(5):1547–1554. 10.1002/hep.27368 25125077

[19] Ekstedt M, Franzen LE, Mathiesen UL, Long-term follow-up of patients with NAFLD and elevated liver enzymes. Hepatology. 2006;44(4):865–873. 10.1002/hep.21327 17006923

[20] Pinyol R, Torrecilla S, Wang H, Molecular characterisation of hepatocellular carcinoma in patients with non-alcoholic steatohepatitis. J Hepatol. 2021;75(4):865–878. 10.1016/j.jhep.2021.04.049 33992698 PMC12164395

[21] Milic S, Lulic D, Stimac D. Non-alcoholic fatty liver disease and obesity: biochemical, metabolic and clinical presentations. World J Gastroenterol. 2014;20(28):9330–9337. 10.3748/wjg.v20.i28.9330 25071327 PMC4110564

[22] Nasereldin DS, White LJ, Hodge DO, Roberts LR, Patel T, Antwi SO. Association of metabolic health phenotypes, obesity, and hepatocellular carcinoma risk. Dig Liver Dis. 2022;54(7):964–972. 10.1016/j.dld.2021.12.002 34953761 PMC9213572

[23] Calle EE, Rodriguez C, Walker-Thermond K, Thun MJ. Overweight, obesity, and mortality from cancer in a prospectively studied cohorts of U.S. adults. N Engl J Med. 2003;348:1625–1638. 12711737 10.1056/NEJMoa021423

[24] Lauby-Secretan B, Scoccianti C, Loomis D, Grosse Y, Bianchini F, Straif K. Body fatness and cancer-viewpoint of the IARC working group. N Engl J Med. 2016;375:794–798. 27557308 10.1056/NEJMsr1606602 PMC6754861

[25] Ratziu V, Giral P, Charlotte F, Liver fibrosis in overweight patients. Gastroenterology. 2000;118:1117–1123. 10.1053/gast.2000.7964 10833486

[26] Parikh ND, Marrero WJ, Wang J, Projected increase in obesity and non-alcoholic-steatohepatitis-related liver transplantation waitlist additions in the United States. Hepatology. 2019;70(2):487–495. 10.1002/hep.29473 28833326

[27] Park EJ, Lee JH, Yu GY, Dietary and genetic obesity promote liver inflammation and tumorigenesis by enhancing IL-6 and TNF expression. Cell. 2010;140(2):197–208. 10.1016/j.cell.2009.12.052 20141834 PMC2836922

[28] Nakagawa H, Umemura A, Taniguchi K, ER stress cooperates with hypernutrition to trigger TNF-dependent spontaneous HCC development. Cancer Cell. 2014;26(3):331–343. 10.1016/j.ccr.2014.07.001 25132496 PMC4165611

[29] Albhaisi S, Chowdhury A, Sanyal AJ. Non-alcoholic fatty liver disease in lean individuals. JHEP Rep. 2019;1(4):329–341. 10.1016/j.jhepr.2019.08.002 32039383 PMC7001558

[30] Zhang K, Kaufman RJ. From endoplasmic-reticulum stress to the inflammatory response. Nature. 2008;454(7203):455–462. 10.1038/nature07203 18650916 PMC2727659

[31] Fu S, Watkins SM, Hotamisligil GS. The role of endoplasmic reticulum in hepatic lipid homeostasis and stress signaling. Cell Metab. 2012;15(5):623–634. 10.1016/j.cmet.2012.03.007 22560215

[32] Cox JS, Shamu CE, Walter P. Transcriptional inductions of genes encoding endoplasmic recticulum resident proteins require a transmembrane protein kinase. Cell. 1993;73:1197–1206. 8513503 10.1016/0092-8674(93)90648-a

[33] Tirasophon W, Welihinda AA, Kaufman RJ. A stress response pathway from the endoplasmic recticulum to the nucleus requires a novel bifunctional protein kinase/endonuclease(ire1p) in mammalian cells. Gene Dev. 1988;12:1812–1824. 10.1101/gad.12.12.1812 PMC316900 9637683

[34] Mori K, Ma W, Gething M, Sambrook J. A transmembrane protein with a cdc2+/CDC28-related kinase activity is required for signaling from the ER to the nucleus. Cell. 1993;74:743–756. 8358794 10.1016/0092-8674(93)90521-q

[35] Kimata Y, Oikawa D, Shimizu Y, Ishiwata-Kimata Y, Kohno K. A role for BiP as an adjustor for the endoplasmic reticulum stress-sensing protein Ire1. J Cell Biol. 2004;167(3):445–456. 10.1083/jcb.200405153 15520230 PMC2172501

[36] Lee AS. The ER chaperone and signaling regulator GRP78/BiP as a monitor of endoplasmic reticulum stress. Methods. 2005;35(4):373–381. 10.1016/j.ymeth.2004.10.010 15804610

[37] Pincus D, Chevalier MW, Aragon T, BiP binding to the ER-stress sensor Ire1 tunes the homeostatic behavior of the unfolded protein response. PLoS Biol. 2010;8(7):e1000415. 10.1371/journal.pbio.1000415 20625545 PMC2897766

[38] Ali MM, Bagratuni T, Davenport EL, Structure of the Ire1 autophosphorylation complex and implications for the unfolded protein response. EMBO J. 2011;30(5):894–905. 10.1038/emboj.2011.18 21317875 PMC3049214

[39] Han D, Lerner AG, Vande Walle L, IRE1alpha kinase activation modes control alternate endoribonuclease outputs to determine divergent cell fates. Cell. 2009;138(3):562–575. 10.1016/j.cell.2009.07.017 19665977 PMC2762408

[40] Lee KP, Dey M, Neculai D, Cao C, Dever TE, Sicheri F. Structure of the dual enzyme Ire1 reveals the basis for catalysis and regulation in nonconventional RNA splicing. Cell. 2008;132(1):89–100. 10.1016/j.cell.2007.10.057 18191223 PMC2276645

[41] Tam AB, Koong AC, Niwa M. Ire1 has distinct catalytic mechanisms for XBP1/HAC1 splicing and RIDD. Cell Rep. 2014;9(3):850–858. 10.1016/j.celrep.2014.09.016 25437541 PMC4486022

[42] Calfon M, Zeng H, Urano F, IRE1 couples endoplasmic reticulum load to secretory capacity by processing the XBP-1 mRNA. Nature. 2002;415:92–96. 11780124 10.1038/415092a

[43] Upton J-P, Wang L, Han D, IRE1*α* cleaves select microRNAs during ER stress to derepress translation of proapoptotic caspase-2. Science. 2012;338(6108):818–822. 10.1126/science.1226191 23042294 PMC3742121

[44] Reimold AM, Iwakoshi NN, Manis J, Plasma cell differentiation requires the transcription factor XBP1. Nature. 2001;421:300–307. 10.1038/35085509 11460154

[45] Lee AH, Iwakoshi NN, Glimcher LH. XBP-1 regulates a subset of endoplasmic reticulum resident chaperone genes in the unfolded protein response. Mol Cell Biol. 2003;23(21):7448–7459. 10.1128/MCB.23.21.7448-7459.2003 14559994 PMC207643

[46] Zhang K, Wang S, Malhotra J, The unfolded protein response transducer IRE1alpha prevents ER stress-induced hepatic steatosis. EMBO J. 2011;30(7):1357–1375. 10.1038/emboj.2011.52 21407177 PMC3094110

[47] Yamamoto K, Sato T, Matsui T, Transcriptional induction of mammalian ER quality control proteins is mediated by single or combined action of ATF6alpha and XBP1. Dev Cell. 2007;13(3):365–376. 10.1016/j.devcel.2007.07.018 17765680

[48] Bommiasamy H, Back SH, Fagone P, ATF6alpha induces XBP1-independent expansion of the endoplasmic reticulum. J Cell Sci. 2009;122(Pt 10):1626–1636. 10.1242/jcs.045625 19420237 PMC2680102

[49] Hassler J, Cao Stewart S, Kaufman Randal J. IRE1, a double-edged sword in pre-miRNA slicing and cell death. Developmental Cell. 2012;23(5):921–923. 10.1016/j.devcel.2012.10.025 23153490 PMC3684431

[50] Yoshida H, Haze K, Yanagi H, Yura T, Mori K. Identification of the cis-acting endoplasmic reticulum stress response element responsible for transcriptional induction of mammalian glucose-regulated proteins. J Biol Chem. 1998;273(50):33741–33749. 10.1074/jbc.273.50.33741 9837962

[51] Haze K, Yoshida H, Yanagi H, Yura T, Mori K. Mammalian transcription factor ATF6 is synthesized as a transmembrane protein and activated by proteolysis in response to endoplasmic reticulum stress. Mol Biol Cell. 1999;10:3787–3799. 10564271 10.1091/mbc.10.11.3787 PMC25679

[52] Shen J, Chen X, Hendershot L, Prywes R. ER stress regulation of ATF6 localization by dissociation of BiP/Grp78 binding and unmasking Golgi localization signal. Developmental Cell. 2002;3:99–111. 12110171 10.1016/s1534-5807(02)00203-4

[53] Ye J, Rawson RB, Komuro R, ER stress induces cleavage of membrane-bound ATF6 by the same protease that process SREBPs. Mol Cell. 2000;6:1355–1364. 11163209 10.1016/s1097-2765(00)00133-7

[54] Gallagher CM, Garri C, Cain EL, Celfins are a new class of unfolded protein response inhibitor, selectively targeting the ATF6α branch. eLife. 2016;5:1–33. 10.7554/eLife.11878.001 PMC4954757 27435960

[55] Yoshida H, Matsui T, Yamamoto A, O’kada T, Mori K. XBP1 mRNA is induced by ATF6 and spliced by IRE1 in response to ER stress to produce highly active trancription factor. Cell. 2001;107:881–891. 11779464 10.1016/s0092-8674(01)00611-0

[56] Fagone P, Jackowski S. Phosphatidylcholine and the CDP-choline cycle. Biochim Biophys Acta. 2013;1831(3):523–532. 10.1016/j.bbalip.2012.09.009 23010477 PMC3562404

[57] Maiuolo J, Bulotta S, Verderio C, Benfante R, Borgese N. Selective activation of the transcription factor ATF6 mediates endoplasmic reticulum proliferation triggered by a membrane protein. Proc Natl Acad Sci USA. 2011;108(19):7832–7837. 10.1073/pnas.1101379108 21521793 PMC3093499

[58] Hardling HP, Zhang Y, Ron D. Protein translation and folding coupled by an endoplasmic reticulum-resident kinase. Nature. 1999;397:271–274. 9930704 10.1038/16729

[59] Chen J, London IM. Regulation of protein synthesis by heme-regulated eIF-2α kinase. Trnas Biochem Sci. 1995;20:105–108. 10.1016/s0968-0004(00)88975-6 7709427

[60] Ron D Translational control in the endoplasmic reticulum stress response. J Clin Invest. 2002;110(10):1383–1388. 10.1172/jci0216784 12438433 PMC151821

[61] Scheuner D, Song B, McEwen E, Translational control id required for the unfolded protein response and in vitro glucose homeostasis. Molecular Cell. 2001;7:1165–1176. 11430820 10.1016/s1097-2765(01)00265-9

[62] Hardling HP, Zeng H, Zhang Y, Diabetes mellitus and exocrine pancreatic dysfunction in PERK−/− mice reveals a role for translational control in secretory cell survival. Molecular Cell. 2001;7:1152–1163. 10.1016/s1097-2765(01)00264-7 11430819

[63] Marciniak SJ, Yun CY, Oyadomari S, CHOP induces death by promoting protein synthesis and oxidation in the stressed endoplasmic reticulum. Genes Dev. Dec 15 2004;18(24):3066–3077. 10.1101/gad.1250704 15601821 PMC535917

[64] Zinszner H, Kuroda M, Wang X, CHOP is implicated in programmed cell death in response to impaired function of the endoplasmic reticulum. Genes Dev. 1998;12:982–995. 9531536 10.1101/gad.12.7.982 PMC316680

[65] Oyadomari S, Mori M. Roles of CHOP/GADD153 in endoplasmic reticulum stress. Cell Death Differ. 2004;11(4):381–389. 10.1038/sj.cdd.4401373 14685163

[66] Novoa I, Zeng H, Harding HP, Ron D. Feedback inhibition of the unfolded protein response by GADD34-mediated dephosphorylation of eIF2. J Cell Biol. 2001;153:1011–1021. 11381086 10.1083/jcb.153.5.1011 PMC2174339

[67] Buzzetti E, Pinzani M, Tsochatzis EA. The multiple-hit pathogenesis of nonalcoholic fatty liver disease (NAFLD). Metabolism. 2016;65(8):1038–1048. 10.1016/j.metabol.2015.12.012 26823198

[68] Weston CR, Davis RJ. The JNK signal transduction pathway. Curr Opin Cell Biol. 2007;19(2):142–149. 10.1016/j.ceb.2007.02.001 17303404

[69] Ly LD, Xu S, Choi SK, Oxidative stress and calcium dysregulation by palmitate in type 2 diabetes. Exp Mol Med. 2017;49(2):e291. 10.1038/emm.2016.157 28154371 PMC5336562

[70] Holzer RG, Park EJ, Li N, Saturated fatty acids induce c-Src clustering within membrane subdomains, leading to JNK activation. Cell. 2011;147(1):173–184. 10.1016/j.cell.2011.08.034 21962514 PMC3295636

[71] Hofmann AF. The Continuing Importance of Bile Acids in Liver and Intestinal Diseases. Arch Intern Med. 1999;159:2647–2658. 10597755 10.1001/archinte.159.22.2647

[72] Ioannou GN, Van Rooyen DM, Savard C, Cholesterol-lowering drugs cause dissolution of cholesterol crystals and disperse Kupffer cell crown-like structures during resolution of NASH. J Lipid Res. 2015;56(2):277–285. 10.1194/jlr.M053785 25520429 PMC4306682

[73] Bruschi FV, Claudel T, Tardelli M, The PNPLA3 I148M variant modulates the fibrogenic phenotype of human hepatic stellate cells. Hepatology. 2017;65(6):1875–1890. 10.1002/hep.29041 28073161

[74] Dongiovanni P, Donati B, Fares R, PNPLA3 I148M polymorphism and progressive liver disease. World J Gastroenterol. 2013;19(41):6969–6978. 10.3748/wjg.v19.i41.6969 24222941 PMC3819533

[75] Jang C, Wada S, Yang S, The small intestine shields the liver from fructose-induced steatosis. Nat Metab. 2020;2(7):586–593. 10.1038/s42255-020-0222-9 32694791 PMC8020332

[76] Todoric J, Di Caro G, Reibe S, Fructose stimulated de novo lipogenesis is promoted by inflammation. Nat Metab. 2020;2(10):1034–1045. 10.1038/s42255-020-0261-2 32839596 PMC8018782

[77] Zhang P, Liu J, Lee A, IL-22 resolves MASLD via enterocyte STAT3 restoration of diet-perturbed intestinal homeostasis. Cell Metab. 2024;36(10):2341–2354.e6. 10.1016/j.cmet.2024.08.012 39317186 PMC11631175

[78] Aron-Wisnewsky J, Vigliotti C, Witjes J, Gut microbiota and human NAFLD: disentangling microbial signatures from metabolic disorders. Nat Rev Gastroenterol Hepatol. 2020;17(5):279–297. 10.1038/s41575-020-0269-9 32152478

[79] Dara L, Ji C, Kaplowitz N. The contribution of endoplasmic reticulum stress to liver diseases. Hepatology. 2011;53(5):1752–1763. 10.1002/hep.24279 21384408 PMC3082587

[80] Donnelly KL, Smith CI, Schwargenburg SJ, Jessurun J, Boldt MD, Parks E. Sources of fatty acids stored in liver and secreted via lipoproteins in patients with nonalcoholic fatty liver disease. J Clin Invest. 2005;115:1343–1351. 10.1172/JCI23621 15864352 PMC1087172

[81] Yen CL, Stone SJ, Koliwad S, Harris C, Farese RV Jr. Thematic review series: glycerolipids. DGAT enzymes and triacylglycerol biosynthesis. J Lipid Res. 2008;49(11):2283–2301. 10.1194/jlr.R800018-JLR200 18757836 PMC3837458

[82] Monetti M, Levin MC, Watt MJ, Dissociation of hepatic steatosis and insulin resistance in mice overexpressing DGAT in the liver. Cell Metabolism. 2007;6:69–78. 10.1016/j.cmet.2007.05.005 17618857

[83] Yamaguchi K, Yang L, McCall S, Inhibiting triglyceride synthesis improves hepatic steatosis but exacerbates liver damage and fibrosis in obese mice with nonalcoholic steatosis. Hepatology. 2007;45:1366–1374. 10.1002/hep.21655 17476695

[84] Chitraju C, Mejhert N, Haas JT, Triglyceride synthesis by DGAT1 protects Adipocytes from Lipid-induced ER stress during Lipolysis. Cell Metabolism. 2012;26:407–418. 10.1016/j.cmet.2017.07.012 PMC6195226 28768178

[85] Listenburger LL, Han X, Lewis SE, Triglyceride accumulation protects against fatty acid-induced lipotoxicity. PNAS. 2003;18:3077–3082. 10.1073/pnas.0630588100 PMC152249 12629214

[86] Fabbrini E, Mohammed BS, Magkos F, Korenblat KM, Patterson BW, Klein S. Alterations in adipose tissue and hepatic lipid kinetics in obese men and women with nonalcoholic fatty liver disease. Gastroenterology. 2008;134(2):424–431. 10.1053/j.gastro.2007.11.038 18242210 PMC2705923

[87] de Almeida IT, Cortez-Pinto H, Fidalgo G, Rodrigues D, Camilo ME. Plasma total and free fatty acids composition in human non-alcoholic steatohepatitis. Clin Nutr. 2002;21(3):219–223. 10.1054/clnu.2001.0529 12127930

[88] Puri P, Wiest MM, Cheung O, The plasma lipidomic signature of nonalcoholic steatohepatitis. Hepatology. 2009;50(6):1827–1838. 10.1002/hep.23229 19937697 PMC5031239

[89] Itoh M, Kato H, Suganami T, Hepatic crown-like structure: a unique histological feature in non-alcoholic steatohepatitis in mice and humans. PLoS One. 2013;8(12):e82163. 10.1371/journal.pone.0082163 24349208 PMC3859576

[90] Koo JH, Lee HJ, Kim W, Kim SG. Endoplasmic reticulum stress in hepatic stellate cells promotes liver fibrosis via PERK-mediated degradation of HNRNPA1 and up-regulation of SMAD2. Gastroenterology. 2016;150(1):181–193.e8. 10.1053/j.gastro.2015.09.039 26435271

[91] Kim JY, Garcia-Carbonell R, Yamachika S, ER stress drives lipogenesis and steatohepatitis via caspase-2 activation of S1P. Cell. 2018;175(1):133–145.e15. 10.1016/j.cell.2018.08.020 30220454 PMC6159928

[92] Brown MS, Goldstein JL. The SREBP pathway: regulation of cholesterol metabolism by proteolysis of a membrane-bound transcription factor. Cell. 1997;89:331–340. 9150132 10.1016/s0092-8674(00)80213-5

[93] Kim JY, Wang LQ, Sladky VC, PIDDosome-SCAP crosstalk controls high-fructose-diet-dependent transition from simple steatosis to steatohepatitis. Cell Metab. 2022;34(10):1548–1560.e6. 10.1016/j.cmet.2022.08.005 36041455 PMC9547947

[94] Wang Q, Zhou H, Bu Q, Role of XBP1 in regulating the progression of non-alcoholic steatohepatitis. J Hepatol. 2022;77(2):312–325. 10.1016/j.jhep.2022.02.031 35292349

[95] Puri P, Mirshahi F, Cheung O, Activation and dysregulation of the unfolded protein response in nonalcoholic fatty liver disease. Gastroenterology. 2008;134(2):568–576. 10.1053/j.gastro.2007.10.039 18082745

[96] Lee A, Scapa EF, Cohen D, Glimcher LH. Regulation of hepatic lipogenesis by the transcription factor XBP1. Science. 2008;320:1492–1496. 18556558 10.1126/science.1158042 PMC3620093

[97] Wang S, Chen Z, Lam V, IRE1alpha-XBP1s induces PDI expression to increase MTP activity for hepatic VLDL assembly and lipid homeostasis. Cell Metab. 2012;16(4):473–486. 10.1016/j.cmet.2012.09.003 23040069 PMC3569089

[98] Seki E, Brenner DA, Karin M. A liver full of JNK: signaling in regulation of cell function and disease pathogenesis, and clinical approaches. Gastroenterology. 2012;143(2):307–320. 10.1053/j.gastro.2012.06.004 22705006 PMC3523093

[99] Wagner EF, Nebreda AR. Signal integration by JNK and p38 MAPK pathways in cancer development. Nat Rev Cancer. 2009;9(8):537–549. 10.1038/nrc2694 19629069

[100] Das M, Garlick DS, Greiner DL, Davis RJ. The role of JNK in the development of hepatocellular carcinoma. Genes Dev. 2011;25(6):634–645. 10.1101/gad.1989311 21406557 PMC3059836

[101] Pal M, Febbraio MA, Lancaster GI. The roles of c-Jun NH2-terminal kinases (JNKs) in obesity and insulin resistance. J Physiol. 2016;594(2):267–279. 10.1113/JP271457 26608096 PMC4713744

[102] Micheau O, Tschopp J. Induction of TNF receptor I-mediated apoptosis via two sequential signaling complexes. Cell. 2003;114(2):181–190. 10.1016/s0092-8674(03)00521-x 12887920

[103] Wajant H, Scheurich P. TNFR1-induced activation of the classical NF-kappaB pathway. FEBS J. 2011;278(6):862–876. 10.1111/j.1742-4658.2011.08015.x 21232017

[104] Liu Z, Hsu H, Goeddel D, Karin M. Dissection of TNF receptor 1 effector functions: JNK activation is not linked to apoptosis while NF-kB activation prevents cell death. Cell. 1996;87:565–576. 8898208 10.1016/s0092-8674(00)81375-6

[105] Urano F, Wang X, Bertolotti A, Coupling of stress in the ER to activation of JNK protein kinases by ER transmembrane kinase IRE1. Science. 2000;287:664–666. 10650002 10.1126/science.287.5453.664

[106] Hu P, Han Z, Couvillon AD, Kaufman RJ, Exton JH. Autocrine tumor necrosis factor alpha links endoplasmic reticulum stress to the membrane death receptor pathway through IRE1alpha-mediated NF-kappaB activation and down-regulation of TRAF2 expression. Mol Cell Biol. 2006;26(8):3071–3084. 10.1128/MCB.26.8.3071-3084.2006 16581782 PMC1446932

[107] Deng J, Lu PD, Zhang Y, Translational repression mediates activation of nuclear factor kappa B by phosphorylated translation initiation factor 2. Mol Cell Biol. 2004;24(23):10161–10168. 10.1128/MCB.24.23.10161-10168.2004 15542827 PMC529034

[108] Moon Y, Liang G, Xie X, The SCAP/SREBP pathway is essential for developing diabetic fatty liver and carbohydrate-induced hyperglycemia in animals. Cell Metab. 2012;15:240–246. 10.1016/j.cmet.2011.12.017 22326225 PMC3662050

[109] Osborne TF, Espenshade PJ. Lipid balance must be just right to prevent development of severe liver damage. J Clin Invest. 2022;132(11):e160326. 10.1172/JCI160326 35642642 PMC9151688

[110] Kawamura S, Matsushita Y, Kurosaki S, Inhibiting SCAP/SREBP exacerbates liver injury and carcinogenesis in murine nonalcoholic steatohepatitis. J Clin Invest. 2022;132(11):e151895. 10.1172/JCI151895 35380992 PMC9151706

[111] Watanabe S, Horie Y, Suzuki A. Hepatocyte-specific Pten-deficient mice as a novel model for nonalcoholic steatohepatitis and hepatocellular carcinoma. Hepatol Res. 2005;33(2):161–166. 10.1016/j.hepres.2005.09.026 16214396

[112] Softic S, Gupta MK, Wang GX, Divergent effects of glucose and fructose on hepatic lipogenesis and insulin signaling. J Clin Invest. 2017;127(11):4059–4074. 10.1172/JCI94585 28972537 PMC5663363

[113] Tu BP, Weissman JS. Oxidative protein folding in eukaryotes: mechanisms and consequences. J Cell Biol. 2004;164(3):341–346. 10.1083/jcb.200311055 14757749 PMC2172237

[114] Kaspar JW, Niture SK, Jaiswal AK. Nrf2:INrf2 (Keap1) signaling in oxidative stress. Free Radic Biol Med. 2009;47(9):1304–1309. 10.1016/j.freeradbiomed.2009.07.035 19666107 PMC2763938

[115] Sarcinelli C, Dragic H, Piecyk M, ATF4-dependent NRF2 transcriptional regulation promotes antioxidant protection during endoplasmic reticulum stress. Cancers (Basel). 2020;12(3):569. 10.3390/cancers12030569 32121537 PMC7139862

[116] Cullinan SB, Zhang D, Hannink M, Arvisais E, Kaufman RJ, Diehl JA. Nrf2 is a direct PERK substrate and effector of PERK-dependent cell survival. Mol Cell Biol. 2003;23(20):7198–7209. 10.1128/MCB.23.20.7198-7209.2003 14517290 PMC230321

[117] Lu K, Alcivar AL, Ma J, NRF2 induction supporting breast cancer cell survival is enabled by oxidative stress-induced DPP3-KEAP1 interaction. Cancer Res. 2017;77(11):2881–2892. 10.1158/0008-5472.CAN-16-2204 28416489 PMC5464605

[118] Milkovic L, Zarkovic N, Saso L. Controversy about pharmacological modulation of Nrf2 for cancer therapy. Redox Biol. 2017;12:727–732. 10.1016/j.redox.2017.04.013 28411557 PMC5393166

[119] Gu L, Zhu Y, Nandi SP, FBP1 controls liver cancer evolution from senescent MASH hepatocytes. Nature. 2025;637(8045):461–469. 10.1038/s41586-024-08317-9 39743585 PMC12168545

[120] He F, Antonucci L, Yamachika S, NRF2 activates growth factor genes and downstream AKT signaling to induce mouse and human hepatomegaly. J Hepatol. 2020;72(6):1182–1195. 10.1016/j.jhep.2020.01.023 32105670 PMC8054878

[121] Nelson JE, Wilson L, Brunt EM, Relationship between the pattern of hepatic iron deposition and histological severity in nonalcoholic fatty liver disease. Hepatology. 2011;53(2):448–457. 10.1002/hep.24038 21274866 PMC3058264

[122] Liu J, Kang R, Tang D. Signaling pathways and defense mechanisms of ferroptosis. FEBS J. 2022;289(22):7038–7050. 10.1111/febs.16059 34092035

[123] Corradini E, Pietrangelo A. Iron and steatohepatitis. J Gastroenterol Hepatol. 2012;27(Suppl 2):42–46. 10.1111/j.1440-1746.2011.07014.x 22320915

[124] Ganz T, Nemeth E. Hepcidin and iron homeostasis. Biochim Biophys Acta. 2012;1823(9):1434–1443. 10.1016/j.bbamcr.2012.01.014 22306005 PMC4048856

[125] Tuohetahuntila M, Spee B, Kruitwagen HS, Role of long-chain acyl-CoA synthetase 4 in formation of polyunsaturated lipid species in hepatic stellate cells. Biochim Biophys Acta. 2015;1851(2):220–230. 10.1016/j.bbalip.2014.12.003 25500141

[126] Toyokuni S. Iron and carcinogenesis: from Fenton reaction to target genes. Redox Rep. 2002;7(4):189–197. 10.1179/135100002125000596 12396663

[127] Tsurusaki S, Tsuchiya Y, Koumura T, Hepatic ferroptosis plays an important role as the trigger for initiating inflammation in nonalcoholic steatohepatitis. Cell Death Dis. 2019;10(6):449. 10.1038/s41419-019-1678-y 31209199 PMC6579767

[128] He F, Zhang P, Liu J, ATF4 suppresses hepatocarcinogenesis by inducing SLC7A11 (xCT) to block stress-related ferroptosis. J Hepatol. 2023;79(2):362–377. 10.1016/j.jhep.2023.03.016 36996941 PMC11332364

[129] Lee YS, Lee DH, Choudry HA, Bartlett DL, Lee YJ. Ferroptosis-induced endoplasmic reticulum stress: cross-talk between ferroptosis and apoptosis. Mol Cancer Res. 2018;16(7):1073–1076. 10.1158/1541-7786.MCR-18-0055 29592897 PMC6030493

[130] Hotamisligil GS. Endoplasmic reticulum stress and the inflammatory basis of metabolic disease. Cell. 2010;140(6):900–917. 10.1016/j.cell.2010.02.034 20303879 PMC2887297

[131] Vecchi C, Montosi G, Zhang K, ER Stress controls iron metabolism through induction of hepcidin. Science. 2009;325:877–880. 19679815 10.1126/science.1176639 PMC2923557

[132] Zhang K, Shen X, Wu J, Endoplasmic reticulum stress activates cleavage of CREBH to induce a systemic inflammatory response. Cell. 2006;124(3):587–599. 10.1016/j.cell.2005.11.040 16469704

[133] Kowdley KV, Belt P, Wilson LA, Serum ferritin is an independent predictor of histologic severity and advanced fibrosis in patients with nonalcoholic fatty liver disease. Hepatology. 2012;55(1):77–85. 10.1002/hep.24706 21953442 PMC3245347

[134] Gautheron J, Vucur M, Reisinger F, A positive feedback loop between RIP3 and JNK controls non-alcoholic steatohepatitis. EMBO Mol Med. 2014;6(8):1062–1074. 10.15252/emmm.201403856 24963148 PMC4154133

[135] Koo GB, Morgan MJ, Lee DG, Methylation-dependent loss of RIP3 expression in cancer represses programmed necrosis in response to chemotherapeutics. Cell Res. 2015;25(6):707–725. 10.1038/cr.2015.56 25952668 PMC4456623

[136] Feldstein AE, Werneburg NW, Canbay A, Free fatty acids promote hepatic lipotoxicity by stimulating TNF-alpha expression via a lysosomal pathway. Hepatology. 2004;40(1):185–194. 10.1002/hep.20283 15239102

[137] Neuschwander-Tetri BA. Hepatic lipotoxicity and the pathogenesis of nonalcoholic steatohepatitis: the central role of nontriglyceride fatty acid metabolites. Hepatology. 2010;52(2):774–788. 10.1002/hep.23719 20683968

[138] Pineau L, Colas J, Dupont S, Lipid-induced ER stress: synergistic effects of sterols and saturated fatty acids. Traffic. 2009;10(6):673–690. 10.1111/j.1600-0854.2009.00903.x 19302420

[139] Salvado L, Coll T, Gomez-Foix AM, Oleate prevents saturated-fatty-acid-induced ER stress, inflammation and insulin resistance in skeletal muscle cells through an AMPK-dependent mechanism. Diabetologia. 2013;56(6):1372–1382. 10.1007/s00125-013-2867-3 23460021

[140] Wang D, Wei Y, Pagliassotti MJ. Saturated fatty acids promote endoplasmic reticulum stress and liver injury in rats with hepatic steatosis. Endocrinology. 2006;147(2):943–951. 10.1210/en.2005-0570 16269465

[141] Gorden DL, Myers DS, Ivanova PT, Biomarkers of NAFLD progression: a lipidomics approach to an epidemic. J Lipid Res. 2015;56(3):722–736. 10.1194/jlr.P056002 25598080 PMC4340319

[142] Feldstein AE, Canbay A, Angulo P, Hepatocyte apoptosis and fas expression are prominent features of human nonalcoholic steatohepatitis. Gastroenterology. 2003;125(2):437–443. 10.1016/s0016-5085(03)00907-7 12891546

[143] Cazanave SC, Elmi NA, Akazawa Y, Bronk SF, Mott JL, Gores GJ. CHOP and AP-1 cooperatively mediate PUMA expression during lipoapoptosis. Am J Physiol Gastrointest Liver Physiol. 2010;299(1):G236–G243. 10.1152/ajpgi.00091.2010 20430872 PMC2904106

[144] Recena Aydos L, Aparecida do Amaral L, Serafim de Souza R, Jacobowski AC, Freitas Dos Santos E, Rodrigues Macedo ML. Nonalcoholic fatty liver disease induced by high-fat diet in C57bl/6 models. Nutrients. Dec 16 2019;11(12):306. 10.3390/nu11123067 31888190 PMC6949901

[145] Febbraio MA, Reibe S, Shalapour S, Ooi GJ, Watt MJ, Karin M. Preclinical models for studying NASH-driven HCC: how useful are they? Cell Metab. 2019;29(1):18–26. 10.1016/j.cmet.2018.10.012 30449681 PMC6326872

[146] Leclercq IA, Farrell GC, Schriemer R, Robertson GR. Leptin is essential for the hepatic fibrotic response to chronic liver injury. J Hepatol. 2002;37:206–213. 12127425 10.1016/s0168-8278(02)00102-2

[147] Lin S, Thomas TC, Storlien LH, Huang XF. Development of high fat diet-induced obesity and leptin resistance in C57BL/6J mice. Int J Obes. 2000;24:639–649. 10.1038/sj.ijo.0801209 10849588

[148] Yang L, Calay ES, Fan J, METABOLISM. S-Nitrosylation links obesity-associated inflammation to endoplasmic reticulum dysfunction. Science. 2015;349(6247):500–506. 10.1126/science.aaa0079 26228140 PMC4573582

[149] Soltis AR, Kennedy NJ, Xin X, Hepatic dysfunction caused by consumption of a high-fat diet. Cell Rep. 2017;21(11):3317–3328. 10.1016/j.celrep.2017.11.059 29241556 PMC5734865

[150] Caballero F, Fernandez A, De Lacy AM, Fernandez-Checa JC, Caballeria J, Garcia-Ruiz C. Enhanced free cholesterol, SREBP-2, and StAR expression in human NASH. J Hepatol. 2009;50:789–796. 10.1016/j.jhep.2008.12.016 19231010

[151] Weglarz TC, Degen JL, Sandgren EP. Hepatocyte transplantation into diseased mouse liver. Kinetics of parenchymal repopulation and identification of the proliferative capacity of tetraploid and octaploid hepatocytes. Am J Pathol. 2000;157(6):1963–1974. 10.1016/S0002-9440(10)64835-3 11106569 PMC1885759

[152] Sandgren EP, Palmiter RD, Heckel JL, Daugherty CC, Brinster RH, L DJ. Complete hepatic regeneration after somatic deletion of an albumin-plasminogen activator transgene. Cell. 1991;66:245–256. 1713128 10.1016/0092-8674(91)90615-6

[153] Collino M High dietary fructose intake: sweet or bitter life? World J Diabetes. 2011;2(6):77–81. 10.4239/wjd.v2.i6.77 21860690 PMC3158875

[154] Chanmugam P, Guthrie JF, Cecilio S, Morton JF, Basiotis P, ANAND R. Did fat intake in the United States really decline between 1989–1991 and 1994–1996? J Am Diabet Assoc. 2003;103:867–872. 10.1053/jada.2003.50166 12830026

[155] Marriott BP, Cole N, Lee E. National estimates of dietary fructose intake increased from 1977 to 2004 in the United States. J Nutr. 2009;139(6):1228S–1235S. 10.3945/jn.108.098277 19403716

[156] Cox CL, Stanhope KL, Schwarz JM, Consumption of fructose- but not glucose-sweetened beverages for 10 weeks increases circulating concentrations of uric acid, retinol binding protein-4, and gamma-glutamyl transferase activity in overweight/obese humans. Nutr Metab. 2012;9(1):58. 10.1186/1743-7075-9-68 PMC3463498 22828276

[157] Le KA, Ith M, Kreis R, Fructose overconsumption causes dyslipidemia and ectopic lipid deposition in healthy subjects with and without a family history of type 2 diabetes. Am J Clin Nutr. 2009;89(6):1760–1765. 10.3945/ajcn.2008.27336 19403641

[158] Abdelmalek MF, Suzuki A, Guy C, Increased fructose consumption is associated with fibrosis severity in patients with nonalcoholic fatty liver disease. Hepatology. 2010;51(6):1961–1971. 10.1002/hep.23535 20301112 PMC2922495

[159] Kumamoto R, Uto H, Oda K, Dietary fructose enhances the incidence of precancerous hepatocytes induced by administration of diethylnitrosamine in rat. Eur J Med Res. 2013;18:1–8. 24321741 10.1186/2047-783X-18-54 PMC4029300

[160] Ebbeling CB, Feldman HA, Chomitz VR, A randomized trial of sugar-sweetened beverages and adolescent body weight. N Engl J Med. 2012;367(15):1407–1416. 10.1056/NEJMoa1203388 22998339 PMC3494993

[161] Heinz F, Lamprecht W, Kirsch J. Enzymes of fructose metabolism in human liver. J Clin Invest. 1968;47(8):1826–1832. 10.1172/JCI105872 4385849 PMC297342

[162] Asipu A, Hayward BE, O’Reilly J, Bouthron DT. Properties of normal and mutant recombinant human ketohexokinases and implications for pathogenesis of essential fructosuria. Diabetes. 2003;52:2426–2432. 12941785 10.2337/diabetes.52.9.2426

[163] Ishimoto T, Lanaspa MA, Le MT, Opposing effects of fructokinase C and A isoforms on fructose-induced metabolic syndrome in mice. Proc Natl Acad Sci USA. 2012;109(11):4320–4325. 10.1073/pnas.1119908109 22371574 PMC3306692

[164] Taniguchi K, Wu LW, Grivennikov SI, A gp130-Src-YAP module links inflammation to epithelial regeneration. Nature. 2015;519(7541):57–62. 10.1038/nature14228 25731159 PMC4447318

[165] Llorente C, Raya Tonetti F, Bruellman R, mAChR4 suppresses liver disease via GAP-induced antimicrobial immunity. Nature. 2025;646(8083):180–189. 10.1038/s41586-025-09395-z 40836099 PMC12991828

[166] Tripathi A, Debelius J, Brenner DA, The gut-liver axis and the intersection with the microbiome. Nat Rev Gastroenterol Hepatol. 2018;15(7):397–411. 10.1038/s41575-018-0011-z 29748586 PMC6319369

[167] Ren LP, Chan SM, Zeng XY, Differing endoplasmic reticulum stress response to excess lipogenesis versus lipid oversupply in relation to hepatic steatosis and insulin resistance. PLoS One. 2012;7(2):e30816. 10.1371/journal.pone.0030816 22355328 PMC3280252

[168] Charlton M, Krishnan A, Viker K, Fast food diet mouse: novel small animal model of NASH with ballooning, progressive fibrosis, and high physiological fidelity to the human condition. Am J Physiol Gastrointest Liver Physiol. 2011;301(5):G825–G834. 10.1152/ajpgi.00145.2011 21836057 PMC3220319

[169] Krishnasamy Y, Gooz M, Lemasters JJ, Zhong Z. Role of mitochondrial depolarization and disrupted mitochondrial homeostasis in non-alcoholic steatohepatitis and fibrosis in mice. Int J Physiol Pharmacol. 2019;11:190–204. PMC6872485 31777643

[170] Ganguly S, Muench GA, Shang L, Nonalcoholic steatohepatitis and HCC in a hyperphagic mouse accelerated by western diet. Cell Mol Gastroenterol Hepatol. 2021;12(3):891–920. 10.1016/j.jcmgh.2021.05.010 34062281 PMC8342972

[171] Asgharpour A, Cazanave SC, Pacana T, A diet-induced animal model of non-alcoholic fatty liver disease and hepatocellular cancer. J Hepatol. 2016;65(3):579–588. 10.1016/j.jhep.2016.05.005 27261415 PMC5012902

[172] Clapper JR, Hendricks MD, Gu G, Diet-induced mouse model of fatty liver disease and nonalcoholic steatohepatitis reflecting clinical disease progression and methods of assessment. Am J Physiol Gastrointest Liver Physiol. 2013;305(7):G483–G495. 10.1152/ajpgi.00079.2013 23886860

[173] AEgidius HM, Veidal SS, Feigh M, Multi-omics characterization of a diet-induced obese model of non-alcoholic steatohepatitis. Sci Rep. 2020;10(1):1148. 10.1038/s41598-020-58059-7 31980690 PMC6981216

[174] Tetri LH, Basaranoglu M, Brunt EM, Yerian LM, Neuschwander-Tetri BA. Severe NAFLD with hepatic necroinflammatory changes in mice fed trans fats and a high-fructose corn syrup equivalent. Am J Physiol Gastrointest Liver Physiol. 2008;295(5):G987–G995. 10.1152/ajpgi.90272.2008 18772365 PMC4059366

[175] Dowman JK, Hopkins LJ, Reynolds GM, Development of hepatocellular carcinoma in a murine model of nonalcoholic steatohepatitis induced by use of a high-fat/fructose diet and sedentary lifestyle. Am J Pathol. 2014;184(5):1550–1561. 10.1016/j.ajpath.2014.01.034 24650559 PMC4005975

[176] Cho EJ, Yoon JH, Kwak MS, Tauroursodeoxycholic acid attenuates progression of steatohepatitis in mice fed a methionine-choline-deficient diet. Dig Dis Sci. 2014;59(7):1461–1474. 10.1007/s10620-014-3217-0 24865256

[177] Matsumoto M, Hada N, Sakamaki Y, An improved mouse model that rapidly develops fibrosis in non-alcoholic steatohepatitis. Int J Exp Pathol. 2013;94(2):93–103. 10.1111/iep.12008 23305254 PMC3607137

[178] Muraki Y, Makita Y, Yamasaki M, Amano Y, Matsuo T. Elevation of liver endoplasmic reticulum stress in a modified choline-deficient l-amino acid-defined diet-fed non-alcoholic steatohepatitis mouse model. Biochem Biophys Res Commun. 2017;486(3):632–638. 10.1016/j.bbrc.2017.03.072 28322783

[179] Ikawa-Yoshida A, Matsuo S, Kato A, Hepatocellular carcinoma in a mouse model fed a choline-deficient, L-amino acid-defined, high-fat diet. Int J Exp Pathol. 2017;98(4):221–233. 10.1111/iep.12240 28895242 PMC5639266

[180] Rinella ME, Tacke F, Sanyal AJ, Anstee QM, participants of the AEW. Report on the AASLD/EASL joint workshop on clinical trial endpoints in NAFLD. J Hepatol. 2019;71(4):823–833. 10.1016/j.jhep.2019.04.019 31300231

[181] Vilar-Gomez E, Martinez-Perez Y, Calzadilla-Bertot L, Weight loss through lifestyle modification significantly reduces features of nonalcoholic steatohepatitis. Gastroenterology. 2015;149(2):367–78 e5; quiz e14–5. 10.1053/j.gastro.2015.04.005 25865049

[182] Alam S, Jahid Hasan M, Khan MAS, Alam M, Hasan N. Effect of weight reduction on histological activity and fibrosis of lean nonalcoholic steatohepatitis patient. J Transl Int Med. 2019;7(3):106–114. 10.2478/jtim-2019-0023 31637181 PMC6795050

[183] Malespin MH, Barritt IAS, Watkins SE, Weight loss and weight regain in usual clinical practice: result from the TARGET-NASH observational cohort. Clin Gastroenterol Hepatol. 2022;20:2393–2395. 10.1016/j.cgh.2021.01.023 33486083

[184] Bruinstroop E, Dalan R, Cao Y, Low-dose levothyroxine reduces intrahepatic lipid content in patients with type 2 diabetes mellitus and NAFLD. J Clin Endocrinol Metab. 2018;103(7):2698–2706. 10.1210/jc.2018-00475 29718334

[185] Harrison SA, Bashir MR, Guy CD, Resmetirom (MGL-3196) for the treatment of non-alcoholic steatohepatitis: a multicentre, randomised, double-blind, placebo-controlled, phase 2 trial. Lancet. 2019;394(10213):2012–2024. 10.1016/S0140-6736(19)32517-6 31727409

[186] Harrison SA, Bedossa P, Guy CD, A phase 3, randomized, controlled trial of resmetirom in NASH with liver fibrosis. N Engl J Med. 2024;390(6):497–509. 10.1056/NEJMoa2309000 38324483

[187] Armstrong MJ, Gaunt P, Aithal GP, Liraglutide safety and efficacy in patients with non-alcoholic steatohepatitis (LEAN): a multicentre, double-blind, randomised, placebo-controlled phase 2 study. Lancet. 2016;387(10019):679–690. 10.1016/S0140-6736(15)00803-X 26608256

[188] Newsome PN, Buchholtz K, Cusi K, A placebo-controlled trial of subcutaneous semaglutide in nonalcoholic steatohepatitis. N Engl J Med. 2021;384(12):1113–1124. 10.1056/NEJMoa2028395 33185364

[189] Loomba R, Abdelmalek MF, Armstrong MJ, Semaglutide 2.4 mg once weekly in patients with non-alcoholic steatohepatitis-related cirrhosis: a randomised, placebo-controlled phase 2 trial. Lancet Gastroenterol Hepatol. 2023;8(6):511–522. 10.1016/S2468-1253(23)00068-7 36934740 PMC10792518

[190] Loomba R, Hartman ML, Lawitz EJ, Tirzepatide for metabolic dysfunction-associated steatohepatitis with liver fibrosis. N Engl J Med. 2024;391(4):299–310. 10.1056/NEJMoa2401943 38856224

[191] Sanyal AJ, Newsome PN, Kliers I, Phase 3 trial of semaglutide in metabolic dysfunction-associated steatohepatitis. N Engl J Med. 2025;392(21):2089–2099. 10.1056/NEJMoa2413258 40305708

[192] Ratziu V, Harrison SA, Francque S, Elafibranor, an agonist of the peroxisome proliferator-activated receptor-alpha and -delta, induces resolution of nonalcoholic steatohepatitis without fibrosis worsening. Gastroenterology. 2016;150(5):1147–1159.e5. 10.1053/j.gastro.2016.01.038 26874076

[193] Francque SM, Bedossa P, Ratziu V, A randomized, controlled trial of the pan-PPAR agonist lanifibranor in NASH. N Engl J Med. 2021;385(17):1547–1558. 10.1056/NEJMoa2036205 34670042

[194] Mudaliar S, Henry RR, Sanyal AJ, Efficacy and safety of the farnesoid X receptor agonist obeticholic acid in patients with type 2 diabetes and nonalcoholic fatty liver disease. Gastroenterology. 2013;145(3):574–582.e1. 10.1053/j.gastro.2013.05.042 23727264

[195] Neuschwander-Tetri BA, Loomba R, Sanyal AJ, Farnesoid X nuclear receptor ligand obeticholic acid for non-cirrhotic, non-alcoholic steatohepatitis (FLINT): a multicentre, randomised, placebo-controlled trial. Lancet. 2015;385(9972):956–965. 10.1016/S0140-6736(14)61933-4 25468160 PMC4447192

[196] Younossi ZM, Ratziu V, Loomba R, Obeticholic acid for the treatment of non-alcoholic steatohepatitis: interim analysis from a multicentre, randomised, placebo-controlled phase 3 trial. Lancet. 2019;394(10215):2184–2196. 10.1016/S0140-6736(19)33041-7 31813633

[197] Younossi ZM, Stepanova M, Nader F, Obeticholic acid impact on quality of life in patients with nonalcoholic steatohepatitis: REGENERATE 18-month interim analysis. Clin Gastroenterol Hepatol. 2022;20(9):2050–2058.e12. 10.1016/j.cgh.2021.07.020 34274514

[198] Sanyal A, Charles ED, Neuschwander-Tetri BA, Pegbelfermin (BMS-986036), a PEGylated fibroblast growth factor 21 analogue, in patients with non-alcoholic steatohepatitis: a randomised, double-blind, placebo-controlled, phase 2a trial. Lancet. 2019;392(10165):2705–2717. 10.1016/S0140-6736(18)31785-9 30554783

[199] Harrison SA, Ruane PJ, Freilich BL, Efruxifermin in non-alcoholic steatohepatitis: a randomized, double-blind, placebo-controlled, phase 2a trial. Nat Med. 2021;27(7):1262–1271. 10.1038/s41591-021-01425-3 34239138

[200] Harrison SA, Ruane PJ, Freilich B, A randomized, double-blind, placebo-controlled phase IIa trial of efruxifermin for patients with compensated NASH cirrhosis. JHEP Rep. 2023;5(1):100563. 10.1016/j.jhepr.2022.100563 36644237 PMC9832280

[201] Harrison SA, Frias JP, Neff G, Safety and efficacy of once-weekly efruxifermin versus placebo in non-alcoholic steatohepatitis (HARMONY): a multicentre, randomised, double-blind, placebo-controlled, phase 2b trial. Lancet Gastroenterol Hepatol. 2023;8(12):1080–1093. 10.1016/S2468-1253(23)00272-8 37802088

[202] Loomba R, Sanyal AJ, Kowdley KV, Randomized, controlled trial of the FGF21 analogue pegozafermin in NASH. N Engl J Med. 2023;389(11):998–1008. 10.1056/NEJMoa2304286 37356033 PMC10718287

[203] Loomba R, Sanyal AJ, Nakajima A, Pegbelfermin in patients with nonalcoholic steatohepatitis and stage 3 fibrosis (FALCON 1): a randomized phase 2b study. Clin Gastroenterol Hepatol. 2024;22(1):102–112.e9. 10.1016/j.cgh.2023.04.011 37088457

[204] Abdelmalek MF, Sanyal AJ, Nakajima A, Pegbelfermin in patients with nonalcoholic steatohepatitis and compensated cirrhosis (FALCON 2): a randomized phase 2b study. Clin Gastroenterol Hepatol. 2024;22(1):113–123.e9. 10.1016/j.cgh.2023.04.012 37088458

[205] Alkhouri N, Lawitz E, Noureddin M, DeFronzo R, Shulman GI. GS-0976 (Firsocostat): an investigational liver-directed acetyl-CoA carboxylase (ACC) inhibitor for the treatment of non-alcoholic steatohepatitis (NASH). Expert Opin Investig Drugs. 2020;29(2):135–141. 10.1080/13543784.2020.1668374 PMC7063378 31519114

[206] Loomba R, Mohseni R, Lucas JK, TVB-2640 (FASN inhibitor) for the treatment of nonalcoholic steatohepatitis: FASCINATE-1, a randomized, placebo-controlled phase 2a trials. Gasetroenterology. 2021;161:1475–1486. 10.1053/j.gastro.2021.07.025 34310978

[207] Loomba R, Bedossa P, Grimmer K, Denifanstat for the treatment of metabolic dysfunction-associated steatohepatitis: a multicentre, double-blind, randomised, placebo-controlled, phase 2b trial. Lancet Gastroenterol Hepatol. 2024;9(12):1090–1100. 10.1016/S2468-1253(24)00246-2 39396529

[208] Koutoukidis DA, Astbury NM, Tudor KE, Association of weight loss interventions with changes in biomarkers of nonalcoholic fatty liver disease: a systematic review and meta-analysis. JAMA Intern Med. 2019;179(9):1262–1271. 10.1001/jamainternmed.2019.2248 31260026 PMC6604126

[209] Koutoukidis DA, Koshiaris C, Henry JA, The effect of the magnitude of weight loss on non-alcoholic fatty liver disease: a systematic review and meta-analysis. Metabolism. 2021;115:154455. 10.1016/j.metabol.2020.154455 33259835

[210] Mullur R, Liu YY, Brent GA. Thyroid hormone regulation of metabolism. Physiol Rev. 2014;94(2):355–382. 10.1152/physrev.00030.2013 24692351 PMC4044302

[211] Jo S, Fonseca TL, Bocco B, Type 2 deiodinase polymorphism causes ER stress and hypothyroidism in the brain. J Clin Invest. 2019;129(1):230–245. 10.1172/JCI123176 30352046 PMC6307951

[212] Schoeller DA. Balancing energy expenditure and body weight. Am J Clin Nutr. 1998;68(4):956S–961S. 10.1093/ajcn/68.4.956S 9771879

[213] Johnstone AM, Murison SD, Duncan JS, Rance KA, Speakman JR. Factors influencing variation in basal metabolic rate include fat-free mass, fat mass, age, and circulating thyroxine but not sex, circulating leptin, or triiodothyronine. Am J Clin Nutr. 2005;82(5):941–948. 10.1093/ajcn/82.5.941 16280423

[214] Lei J, Nowbar S, Mariash CN, Ingbar DM. Thyroid hormone stimulates Na-K- ATPase activity and its plasma membrane insertion in rat alveolar epithelial cell. Am J Physiol Lung Cell Mol Physiol. 2003;285:L762–L772. 12740220 10.1152/ajplung.00376.2002

[215] Chouchani ET, Kazak L, Spiegelman BM. New advances in adaptive thermogenesis: UCP1 and beyond. Cell Metab. 2019;29(1):27–37. 10.1016/j.cmet.2018.11.002 30503034

[216] Bianco AC, Sheng XY, Silva JE. Triiodothyronine amplifies norepinephrine stimulation of uncoupling protein gene transcription by a mechanism not requiring protein synthesis. J Biol Chem. 1988;263(34):18168–18175. 10.1016/s0021-9258(19)81340-6 3192531

[217] Ribeiro MO, Carvalho SD, Schultz JJ, Thyroid hormone–sympathetic interaction and adaptive thermogenesis are thyroid hormone receptor isoform–specific. J Clin Invest. 2001;108(1):97–105. 10.1172/jci200112584 11435461 PMC209342

[218] Lee JY, Takahashi N, Yasubuchi M, Triiodothyronine induces UCP-1 expression and mitochondrial biogenesis in human adipocytes. Am J Physiol Cell Physiol. 2012;302(2):C463–C472. 10.1152/ajpcell.00010.2011 22075692

[219] Shin DJ, Osborne TF. Thyroid hormone regulation and cholesterol metabolism are connected through Sterol Regulatory Element-Binding Protein-2 (SREBP-2). J Biol Chem. 2003;278(36):34114–34118. 10.1074/jbc.M305417200 12829694

[220] Huuskonen J, Vishuu M, Pullinger CR, Fielding PE, Fielding CJ. Regulation of ATP-binding cassette transporter A1 by thyroid hormone receptor. Biochemistry. 2004;43:1626–1632. 14769039 10.1021/bi0301643

[221] Rasmussen BB, Holmbaäck UC, Volpi E, Morio-Liondore B, Paddon-Jones D, Wolfe RR. Malonyl coenzyme A and the regulation of functional carnitine palmitoyltransferase-1 activity and fat oxidation in human skeletal muscle. J Clin Invest. 2002;110(11):1687–1693. 10.1172/jci200215715 12464674 PMC151631

[222] Yin L, Zhang Y, Hillgartner FB. Sterol regulatory element-binding protein-1 interacts with the nuclear thyroid hormone receptor to enhance acetyl-CoA carboxylase-alpha transcription in hepatocytes. J Biol Chem. 2002;277(22):19554–19565. 10.1074/jbc.M111771200 11907029

[223] Martinez-Sanchez N, Seoane-Collazo P, Contreras C, Hypothalamic AMPK-ER stress-JNK1 axis mediates the central actions of thyroid hormones on energy balance. Cell Metab. 2017;26(1):212–229.e12. 10.1016/j.cmet.2017.06.014 28683288 PMC5501726

[224] Zhou L, Ding S, Li Y, Endoplasmic reticulum stress may play a pivotal role in lipid metabolic disorders in a novel mouse model of subclinical hypothyroidism. Sci Rep. 2016;6:31381. 10.1038/srep31381 27539723 PMC4990971

[225] Chung GE, Kim D, Kim W, Non-alcoholic fatty liver disease across the spectrum of hypothyroidism. J Hepatol. 2012;57(1):150–156. 10.1016/j.jhep.2012.02.027 22425701

[226] Liu YY, Schultz JJ, Brent GA. A thyroid hormone receptor alpha gene mutation (P398H) is associated with visceral adiposity and impaired catecholamine- stimulated lipolysis in mice. J Biol Chem. 2003;278(40):38913–38920. 10.1074/jbc.M306120200 12869545

[227] Zhou J, Tripathi M, Ho JP, Thyroid hormone decreases hepatic steatosis, inflammation, and fibrosis in a dietary mouse model of nonalcoholic steato- hepatitis. Thyroid. 2022;32(6):725–738. 10.1089/thy.2021.0621 35317606

[228] Eron MD, Cable EE, Ito BR, Targeting thyroid receptor-beta agonists to the liver reduces cholesterol and triglycerides and improves the therapeutic index. Proc Natl Acad Sci USA. 2007;104:15490–15495. 17878314 10.1073/pnas.0702759104 PMC1978486

[229] Kannt A, Wohlfart P, Madsen AN, Veidal SS, Feigh M, Schmoll D. Activation of thyroid hormone receptor-beta improved disease activity and metabolism independent of body weight in a mouse model of non-alcoholic steatohe- patitis and fibrosis. Br J Pharmacol. 2021;178(12):2412–2423. 10.1111/bph.15427 33655500

[230] Jiang Y, Wang Z, Ma B, GLP-1 improves adipocyte insulin sensitivity following induction of endoplasmic recticulum stress. Front Pharmacol. 2018;9:1–10. 10.3389/fphar.2018.01168 30459598 PMC6232689

[231] Ao N, Yang J, Wang x, Du J, Glucagon-like peptide-1 preserves non-alcoholic fatty liver disease through inhibition of the endoplasmic reticulum stress-associated pathway. Hepatol Res. 2016;46:343–353. 10.1111/hepr.12551 26147696

[232] Buteau J, Roduit R, Susini S, Prentki M. Glucagon-like peptide-1 promotes DNA synthesis, activates phosphatidylinositol-3-kinase and increases transcription factor pancreatic duodenal homeobox gene 1 (PDX-1) DNA binding activity in beta (INS-1) cells. Disbetologia. 1999;42:856–864. 10.1007/s001250051238 10440129

[233] Pontes-da-Silva RM, de Souza Marinho T, de Macedo Cardoso LE, Mandarim- de-Lacerda CA, Aguila MB. Obese mice weight loss role on nonalcoholic fatty liver disease and endoplasmic reticulum stress treated by a GLP-1 receptor agonist. Int J Obes (Lond). 2022;46(1):21–29. 10.1038/s41366-021-00955-7 34465857

[234] MacDonald PE, EL-kholy W, Riedel MJ, Salapatek AMF, Light PE, Wheeler MB. The multiple actions of GLP-1 on the process of glucose-stimulated insulin secretion. Diabetes. 2002;31:S434–S442. 10.2337/diabetes.51.2007.s434 12475787

[235] Holst JJ. The physiology of glucagon-like peptide 1. Physiol Rev. 2007;87(4):1409–1439. 10.1152/physrev.00034.2006 17928588

[236] Kim KS, Hwang E, Park MJ, GLP-q increases preingestive satiation via hypothalamic circuits in mice and humans. Science. 2024;385:438–446. 38935778 10.1126/science.adj2537 PMC11961025

[237] Barritt ASt, Marshman E, Noureddin M. Review article: role of glucagon-like peptide-1 receptor agonists in non-alcoholic steatohepatitis, obesity and diabetes-what hepatologists need to know. Aliment Pharmacol Ther. 2022;55(8):944–959. 10.1111/apt.16794 35266164 PMC9310586

[238] Mirza AZ, Althagafi II, Shamshad H. Role of PPAR receptor in different diseases and their ligands: physiological importance and clinical implications. Eur J Med Chem. 2019;166:502–513. 10.1016/j.ejmech.2019.01.067 30739829

[239] Su Q, Baker C, Christian P, Hepatic mitochondrial and ER stress induced by defective PPARalpha signaling in the pathogenesis of hepatic steatosis. Am J Physiol Endocrinol Metab. 2014;306(11):E1264–E1273. 10.1152/ajpendo.00438.2013 24735884 PMC4280162

[240] Han KL, Choi JS, Lee JY, Therapeutic potential of peroxisome proliferators–activated receptor-alpha/gamma dual agonist with alleviation of endoplasmic reticulum stress for the treatment of diabetes. Diabetes. 2008;57(3):737–745. 10.2337/db07-0972 18065517

[241] Chan SM, Sun RQ, Zeng XY, Activation of PPARalpha ameliorates hepatic insulin resistance and steatosis in high fructose-fed mice despite increased endoplasmic reticulum stress. Diabetes. 2013;62(6):2095–2105. 10.2337/db12-1397 23349482 PMC3661626

[242] Henriksson E, Andersen B. FGF19 and FGF21 for the treatment of NASH-two sides of the same coin? Differential and overlapping effects of FGF19 and FGF21 from mice to human. Front Endocrinol (Lausanne). 2020;11:601349. 10.3389/fendo.2020.601349 33414764 PMC7783467

[243] Wang N, Zou Q, Xu J, Zhang J, Liu J. Ligand binding and heterodimerization with retinoid X receptor alpha (RXRalpha) induce farnesoid X receptor (FXR) conformational changes affecting coactivator binding. J Biol Chem. 2018;293(47):18180–18191. 10.1074/jbc.RA118.004652 30275017 PMC6254343

[244] Kir S, Kliewer SA, Mangelsdorf DJ. Roles of FGF19 in liver metabolism. Cold Spring Harb Symp Quant Biol. 2011;76:139–144. 10.1101/sqb.2011.76.010710 21813638

[245] Sinai CJ, Tohken M, Miyata M, Ward JM, Lambert G, Gonzalez FJ. Targeted disruption of the nuclear receptor FXR/BAR impaired bile acid and lipid homeostasis. Cell. 2000;102:731–744. 11030617 10.1016/s0092-8674(00)00062-3

[246] Davis RA, Miyake JH, Hui TY, Spann NJ. Regulation of cholesterol-7α-hy- droxylase: BAREly missing a SHP. J Lipid Res. 2002;43(4):533–543. 10.1016/s0022-2275(20)31482-6 11907135

[247] Chiang JL, Kimmel R, Stroup D. Regulation of cholesterol 7α-hydroxylase gene (CYP7A1) transcription by the liver orphannuclear receptor (LXRα). Gene. 2001;262:257–265. 11179691 10.1016/s0378-1119(00)00518-7

[248] Goodwin B, Jones SA, Price RR, A regulatory cascade of the nuclear receptors, FXR, SHP-1, and LRH-1 represses bile acid synthesis. Molecular Cell. 2000;6:517–526. 11030332 10.1016/s1097-2765(00)00051-4

[249] Del Castillo-Olivares A, Gil G. Role of FXR and FTF in bile acid-mediated suppression of cholesterol 7α-hydroxylase transcription. Nucl Acid Res. 2000;28:3587–3597. 10.1093/nar/28.18.3587 PMC110735 10982880

[250] Zhang M, Chiang JY. Transcriptional regulation of the human sterol 12alpha-hydroxylase gene (CYP8B1): roles of hepatocyte nuclear factor 4alpha in mediating bile acid repression. J Biol Chem. 2001;276(45):41690–41699. 10.1074/jbc.M105117200 11535594

[251] Li H, Chen F, Shang Q, FXR-activating ligands inhibit rabbit ASBT expression via FXR-SHP-FTF cascade. Am J Physiol Gastrointest Liver Physiol. 2005;288(1):G60–G66. 10.1152/ajpgi.00170.2004 15591588

[252] Boyer JL, Trauner M, Mennone A, Upregulation of a basolateral FXR-dependent bile acid efflux transporter OSTalpha-OSTbeta in cholestasis in humans and rodents. Am J Physiol Gastrointest Liver Physiol. 2006;290(6):G1124–G1130. 10.1152/ajpgi.00539.2005 16423920

[253] Song CS, Echchgadda I, Baek BS, Dehydroepiandrosterone sulfotransferase gene induction by bile acid activated farnesoid X receptor. J Biol Chem. 2001;276(45):42549–42556. 10.1074/jbc.M107557200 11533040

[254] Barbier O, Torra IP, Sirvent A, FXR induces the UGT2B4 enzyme in hepatocytes: a potential mechanism of negative feedback control of FXR activity. Gastroenterology. 2003;124(7):1926–1940. 10.1016/s0016-5085(03)00388-3 12806625

[255] Han CY, Rho HS, Kim A, FXR inhibits endoplasmic reticulum stress- induced NLRP3 inflammasome in hepatocytes and ameliorates liver injury. Cell Rep. 2018;24(11):2985–2999. 10.1016/j.celrep.2018.07.068 30208322

[256] Clifford BL, Sedgeman LR, Williams KJ, FXR activation protects against NAFLD via bile-acid-dependent reductions in lipid absorption. Cell Metab. Aug 3 2021;33(8):1671–1684.e4. 10.1016/j.cmet.2021.06.012 PMC8353952 34270928

[257] Watanabe M, Houten SM, Wang L, Bile acids lower triglyceride levels via a pathway involving FXR, SHP, and SREBP-1c. J Clin Invest. 2004;113(10):1408–1418. 10.1172/JCI21025 15146238 PMC406532

[258] Fang S, Suh JM, Reilly SM, Intestinal FXR agonism promotes adipose tissue browning and reduces obesity and insulin resistance. Nat Med. 2015;21(2):159–165. 10.1038/nm.3760 25559344 PMC4320010

[259] Zhou M, Learned RM, Rossi SJ, DePaoli AM, Tian H, Ling L. Engineered FGF19 eliminates bile acid toxicity and lipotoxicity leading to resolution of stea-tohepatitis and fibrosis in mice. Hepatol Commun. 2017;1(10):1024–1042. 10.1002/hep4.1108 29404440 PMC5721409

[260] Gu M, Zhao P, Zhang S, Betulinic acid alleviates endoplasmic reticulum stress-mediated nonalcoholic fatty liver disease through activation of farnesoid X receptors in mice. Br J Pharmacol. Apr 2019;176(7):847–863. 10.1111/bph.14570 30635917 PMC6433649

[261] Polyzos SA, Kountouras J, Mantzoros CS. Obeticholic acid for the treatment of nonalcoholic steatohepatitis: expectations and concerns. Metabolism. 2020;104:154144. 10.1016/j.metabol.2020.154144 31930974

[262] Alkhouri N, Herring R, Kabler H, Safety and efficacy of combination therapy with semaglutide, cilofexor and firsocostat in patients with non-alcoholic steatohepatitis: A randomised, open-label phase II trial. J Hepatol. 2022;77(3):607–618. 10.1016/j.jhep.2022.04.003 35439567

[263] Powers CJ, Mcleskey SW, Wellstein A. Fibroblast growth factors, their receptors and signaling. Endocr Relat Cancer. 2000;7:165–197. 11021964 10.1677/erc.0.0070165

[264] Kir S, Beddow SA, Samuel VT, FGF19 as a postprandial, insulin-independent activator of hepatic protein and glycogen synthesis. Science. 2011;331:1621–1624. 21436455 10.1126/science.1198363 PMC3076083

[265] Eren F, Kurt R, Ermis F, Atug O, Imeryuz N, Yilmaz Y. Preliminary evidence of a reduced serum level of fibroblast growth factor 19 in patients with biopsy-proven nonalcoholic fatty liver disease. Clin Biochem. 2012;45(9):655–658. 10.1016/j.clinbiochem.2012.03.019 22465275

[266] Schreuder TC, Marsman HA, Lenicek M, The hepatic response to FGF19 is impaired in patients with nonalcoholic fatty liver disease and insulin resistance. Am J Physiol Gastrointest Liver Physiol. 2010;298(3):G440–G445. 10.1152/ajpgi.00322.2009 20093562

[267] Shimizu M, Li J, Maruyama R, Inoue J, Sato R. FGF19 (fibroblast growth factor 19) as a novel target gene for activating transcription factor 4 in response to endoplasmic reticulum stress. Biochem J. 2013;450(1):221–229. 10.1042/BJ20121393 23205607

[268] Teng Y, Zhao H, Gao L, Zhang W, Shull AY, Shay C. FGF19 protects hepatocellular carcinoma cells against endoplasmic reticulum stress via activation of FGFR4-GSK3beta-Nrf2 signaling. Cancer Res. 2017;77(22):6215–6225. 10.1158/0008-5472.CAN-17-2039 28951455

[269] He CH, Gong P, Hu B, Identification of activating transcription factor 4 (ATF4) as an Nrf2-interacting protein. Implication for heme oxygenase-1 gene regulation. J Biol Chem. 2001;276(24):20858–20865. 10.1074/jbc.M101198200 11274184

[270] Kharitonenkov A, Shiyanova TL, Koester A, FGF-21 as a novel metabolic regulator. J Clin Invest. 2005;115(6):1627–1635. 10.1172/JCI23606 15902306 PMC1088017

[271] Badman MK, Pissios P, Kennedy AR, Koukos G, Flier JS, Maratos-Flier E. Hepatic fibroblast growth factor 21 is regulated by PPARalpha and is a key mediator of hepatic lipid metabolism in ketotic states. Cell Metab. 2007;5(6):426–437. 10.1016/j.cmet.2007.05.002 17550778

[272] Schlein C, Talukdar S, Heine M, FGF21 lowers plasma triglycerides by accelerating lipoprotein catabolism in white and brown adipose tissues. Cell Metab. 2016;23(3):441–453. 10.1016/j.cmet.2016.01.006 26853749

[273] Wente W, Efanov AM, Brenner M, Fibroblast growth factor-21 improves pancreatic beta-cell function and survival by activation of extracellular signal-regulated kinase 1/2 and Akt signaling pathways. Diabetes. 2006;55(9):2470–2478. 10.2337/db05-1435 16936195

[274] Ge X, Chen C, Hui X, Wang Y, Lam KS, Xu A. Fibroblast growth factor 21 induces glucose transporter-1 expression through activation of the serum response factor/Ets-like protein-1 in adipocytes. J Biol Chem. 2011;286(40):34533–34541. 10.1074/jbc.M111.248591 21846717 PMC3186365

[275] Arner P, Pettersson A, Mitchell PJ, Dunbar JD, Kharitonenkov A, Ryden M. FGF21 attenuates lipolysis in human adipocytes―a possible link to improved insulin sensitivity. FEBS Lett. 2008;582(12):1725–1730. 10.1016/j.febslet.2008.04.038 18460341

[276] Bookout AL, de Groot MH, Owen BM, FGF21 regulates metabolism and circadian behavior by acting on the nervous system. Nat Med. 2013;19(9):1147–1152. 10.1038/nm.3249 23933984 PMC3769420

[277] Hondares E, Rosell M, Gonzalez FJ, Giralt M, Iglesias R, Villarroya F. Hepatic FGF21 expression is induced at birth via PPARalpha in response to milk intake and contributes to thermogenic activation of neonatal brown fat. Cell Metab. 2010;11(3):206–212. 10.1016/j.cmet.2010.02.001 20197053 PMC2847690

[278] Fisher FM, Kleiner S, Douris N, FGF21 regulates PGC-1alpha and browning of white adipose tissues in adaptive thermogenesis. Genes Dev. 2012;26(3):271–281. 10.1101/gad.177857.111 22302939 PMC3278894

[279] De Sousa-Coelho AL, Relat J, Hondares E, FGF21 mediates the lipid metabolism response to amino acid starvation. J Lipid Res. 2013;54(7):1786–1797. 10.1194/jlr.M033415 23661803 PMC3679382

[280] Jiang S, Yan C, Fang QC, Fibroblast growth factor 21 is regulated by the IRE1alpha-XBP1 branch of the unfolded protein response and counteracts endoplasmic reticulum stress-induced hepatic steatosis. J Biol Chem. 2014;289(43):29751–29765. 10.1074/jbc.M114.565960 25170079 PMC4207989

[281] Yilmaz Y, Eren F, Yonal O, Increased serum FGF21 levels in patients with nonalcoholic fatty liver disease. Eur J Clin Invest. 2010;40(10):887–892. 10.1111/j.1365-2362.2010.02338.x 20624171

[282] Yang c, Lu W, Lin T, Activation of Liver FGF21 in hepatocarcinogenesis and during hepatic stress. BMC Gastroenterol. 2012;13:1–14. 10.1186/1471-230X-13-67 PMC3637159 23590285

[283] Li H, Fang Q, Gao F, Fibroblast growth factor 21 levels are increased in nonalcoholic fatty liver disease patients and are correlated with hepatic triglyceride. J Hepatol. 2010;53(5):934–940. 10.1016/j.jhep.2010.05.018 20675007

[284] Wan XS, Lu XH, Xiao YC, ATF4- and CHOP-dependent induction of FGF21 through endoplasmic reticulum stress. Biomed Res Int. 2014;2014:807874. 10.1155/2014/807874 24900988 PMC4037570

[285] Schaap FG, Kremer AE, Lamers WH, Jansen PL, Gaemers IC. Fibroblast growth factor 21 is induced by endoplasmic reticulum stress. Biochimie. 2013;95(4):692–699. 10.1016/j.biochi.2012.10.019 23123503

[286] Iizuka K, Takeda J, Horikawa Y. Glucose induces FGF21 mRNA expression through ChREBP activation in rat hepatocytes. FEBS Lett. 2009;583(17):2882–2886. 10.1016/j.febslet.2009.07.053 19660458

[287] Kan M, Wu X, Wang F, McKeehan WL. Specificity for fibroblast growth factors determined by heparan sulfate in a binary complex with the receptor kinase. J Biol Chem. 1999;274(22):15947–15952. 10.1074/jbc.274.22.15947 10336501

[288] Kurosu H, Choi M, Ogawa Y, Tissue-specific expression of betaKlotho and fibroblast growth factor (FGF) receptor isoforms determines metabolic activity of FGF19 and FGF21. J Biol Chem. 2007;282(37):26687–26695. 10.1074/jbc.M704165200 17623664 PMC2496965

[289] Huang X, Yang C, Jin C, Luo Y, Wang F, McKeehan WL. Resident hepatocyte fibroblast growth factor receptor 4 limits hepatocarcinogenesis. Mol Carcinog. 2009;48(6):553–562. 10.1002/mc.20494 19009564 PMC2712883

[290] Lan T, Morgan DA, Rahmouni K, FGF19, FGF21, and an FGFR1/betaklotho-activating antibody act on the nervous system to regulate body weight and glycemia. Cell Metab. 2017;26(5):709–718.e3. 10.1016/j.cmet.2017.09.005 28988823 PMC5679468

[291] Mao J, DeMayo F, Li H, Liver-specific deletion of acetyl-CoA carboxylase 1 reduces hepatic triglyceride accumulation without affecting glucose homeostasis. Proc Nat Acad Sci USA. 2006;103:8552–8557. 16717184 10.1073/pnas.0603115103 PMC1570106

[292] Loomba R, Kayali Z, Noureddin M, GS-0976 reduces hepatic steatosis and fibrosis markers in patients with nonalcoholic fatty liver disease. Gastroenterology. 2018;155(5):1463–1473.e6. 10.1053/j.gastro.2018.07.027 30059671 PMC6318218

[293] Amin NB, Darekar A, Anstee QM, Efficacy and safety of an orally administered DGAT2 inhibitor alone or coadministered with a liver-targeted ACC inhibitor in adults with non-alcoholic steatohepatitis (NASH): rationale and design of the phase II, dose-ranging, dose-finding, randomised, placebo-controlled MIRNA (Metabolic Interventions to Resolve NASH with fibrosis) study. BMJ Open. 2022;12(3):e056159. 10.1136/bmjopen-2021-056159 PMC8968568 35354614

[294] Davison BA, Harrison SA, Cotter G, Suboptimal reliability of liver biopsy evaluation has implications for randomized clinical trials. J Hepatol. 2020;73(6):1322–1332. 10.1016/j.jhep.2020.06.025 32610115

[295] Brunt EM, Clouston AD, Goodman Z, Complexity of ballooned hepatocyte feature recognition: Defining a training atlas for artificial intelligence-based imaging in NAFLD. J Hepatol. 2022;76(5):1030–1041. 10.1016/j.jhep.2022.01.011 35090960 PMC10544770

[296] Iyer JS, Juyal D, Le Q, AI-based automation of enrollment criteria and endpoint assessment in clinical trials in liver diseases. Nat Med. 2024;30(10):2914–2923. 10.1038/s41591-024-03172-7 39112795 PMC11485234

[297] Pulaski H, Harrison SA, Mehta SS, Clinical validation of an AI-based pathology tool for scoring of metabolic dysfunction-associated steatohepatitis. Nat Med. 2025;31(1):315–322. 10.1038/s41591-024-03301-2 39496972 PMC11750710

[298] Ratziu V, Francque S, Behling CA, Artificial intelligence scoring of liver biopsies in a phase II trial of semaglutide in nonalcoholic steatohepatitis. Hepatology. 2024;80(1):173–185. 10.1097/HEP.0000000000000723, 1. 38112484 PMC11185915

[299] Cronan JE, Gelmann EP. Physical properties of membrane lipids: Biological relevance and regulation. Bacteriol Rev. 1975;39:232–256. 1100043 10.1128/br.39.3.232-256.1975 PMC413917

[300] Jin C, Felli E, Lange NF, Berzigotti A, Gracia-Sancho J, Dufour JF. Endoplasmic reticulum and mitochondria contacts correlate with the presence and severity of NASH in humans. Int J Mol Sci. 2022;23(15):8348. 10.3390/ijms23158348 35955482 PMC9369179

[301] Arruda AP, Pers BM, Parlakgul G, Guney E, Inouye K, Hotamisligil GS. Chronic enrichment of hepatic endoplasmic reticulum-mitochondria contact leads to mitochondrial dysfunction in obesity. Nat Med. 2014;20(12):1427–1435. 10.1038/nm.3735 25419710 PMC4412031

[302] Ng SWK, Rouhani FJ, Brunner SF, Convergent somatic mutations in metabolism genes in chronic liver disease. Nature. 2021;598(7881):473–478. 10.1038/s41586-021-03974-6 34646017

[303] Zhu M, Lu T, Jia Y, Somatic mutations increase hepatic clonal fitness and regeneration in chronic liver disease. Cell. 2019;177(3):608–621.e12. 10.1016/j.cell.2019.03.026 30955891 PMC6519461

[304] Hatch EM, Fischer AH, Deerinck TJ, Hetzer MW. Catastrophic nuclear envelope collapse in cancer cell micronuclei. Cell. 2013;154(1):47–60. 10.1016/j.cell.2013.06.007 23827674 PMC3749778

[305] Liu K, Yang L, Wang G, Metabolic stress drives sympathetic neuropathy within the liver. Cell Metab. 2021;33(3):666–675.e4. 10.1016/j.cmet.2021.01.012 33545051

[306] Brandt C, Nolte H, Henschke S, Food perception primes hepatic ER homeostasis via melanocortin-dependent control of mTOR activation. Cell. 2018;175(5):1321–1335.e20. 10.1016/j.cell.2018.10.015 30445039 PMC6541012

